# Optimizing Protein Bars With Whey Protein Isolate, Pea Protein Isolate, and Blue Whiting ( *Micromesistius poutassou* ) Fish Protein Hydrolysate: A Simplex‐Centroid Mixture Design Study

**DOI:** 10.1002/fsn3.4701

**Published:** 2024-12-19

**Authors:** Diako Khodaei, Francesco Noci, Lisa Ryan

**Affiliations:** ^1^ School of Food Science and Environmental Health, Environmental Sustainability and Health Institute Technological University Dublin Dublin Ireland; ^2^ Atlantic Technological University Galway Ireland

**Keywords:** bar hardening, fish protein hydrolysate, nutritional value, protein bar, sensory analysis

## Abstract

The aim of this study was to use blue whiting fish protein hydrolysate (BWFPH) as a novel dietary amino acid supplement in whey protein isolate (WPI) and pea protein isolate (PPI)‐based protein bars. The findings indicate that incorporating BWFPH significantly influenced the nutritional profile of the protein bars, leading to a ~93% reduction in hardness compared to bars without the hydrolysate. Additionally, BWFPH effectively delayed the hardening process during storage. However, sensory evaluation revealed that the inclusion of BWFPH adversely affected the sensory characteristics, indicating a need for optimization. Using a simplex centroid mixture design, we identified an optimized protein blend compromising 76.02% WPI, 6.72% PPI, and 17.26% BWPFH, which achieved a balance of improved texture and sensory appeal. These results suggest that high‐protein bars can be enhanced by integrating novel protein sources through careful formulation and optimization.

## Graphical abstract

Blue whiting fish protein hydrolysates (BWFPH) effectively delay protein bar hardening, enhancing product longevity. Employing experimental design techniques optimizes the formulation of protein bars for improved texture and sensory appeal. Protein bars comprising 76% whey protein isolate (WPI), 6.7% pea protein isolate (PPI), and 17.3% BWFPH demonstrate optimal texture and sensory properties.

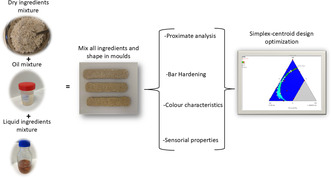

## 1 Introduction

Protein bars have gained significant popularity as convenient and nutritious snacks among different groups of consumers, including individuals looking for a meal replacement or an added nutritional boost for their athletic performance (Burrington 2007). As the market for high‐protein bars is rapidly growing, new formulations are emerging to address consumers' preferences (Sparkman, Smith, and Joyner 2020). However, formulating protein bars can be challenging due to various processing issues that may affect the shape and texture of the bars, including sticking materials, equipment clogging, and cold flow (Keefer et al. 2020). The ratio and type of protein are critical factors that need to be considered before launching a new high‐protein product.

Whey is a byproduct of dairy processing and contains different proteins such as β‐lactoglobulin, α‐lactalbumin, bovine serum albumin, immunoglobulins, and cryoglobulins (Brady 2013). Whey proteins are commonly used in the formulation of protein bars in various forms, including whey protein concentrates, isolates, and hydrolysates. Vegan proteins, like pulse proteins from peas, beans, and lentils, are considered alternatives to whey proteins and can contribute to expanding the consumer market. However, some vegan proteins, such as peanut or soy proteins, contain allergens that limit their use in food production. Other studies have also highlighted that the protein efficiency ratio and biological value of peanuts and soy protein are significantly lower than those of whey proteins (Hoffman and Falvo 2004). Using pulse proteins such as peas, beans, and lentils has been reported as an alternative for allergen‐containing proteins. The protein content in peas is reported to be between 23% and 30%, which exhibits a wide range of functional properties, such as fat binding, foaming, and gelation (Boye, Zare, and Pletch 2010).

Blue whiting ( *Micromesistius poutassou* ) is one of the most prevalent fish species in the Northeast Atlantic. However, despite its abundance, it is often underutilized, primarily being processed into fish oil or fish meals. Research findings suggest blue whiting as a cost‐effective and environmentally sustainable protein source owing to its significant content of high‐quality protein and essential amino acids (Shekoohi et al. 2023). Enzymatic hydrolysis of fish proteins is regarded as a beneficial technique that enhances the bioavailability, bioactivity, and physicochemical characteristics when compared to unprocessed proteins (Egerton et al. 2018). Recent studies suggest that the peptides derived from blue whiting fish protein hydrolysates (BWFPH) have the potential to enhance satiety levels (Cudennec et al. 2012; Heffernan et al. 2022) or exhibit antidiabetic properties (Harnedy‐Rothwell et al. 2021). Nevertheless, incorporating proteins and hydrolysates from fish sources poses a significant hurdle in food product development due to their negative impact on flavor and aroma. In our recent study, it was noted that the addition of BWFPH, up to 10% w/w, improved the protein content and nutritional value of pasta without adverse effects on the sensory attributes or cooking characteristics (Khodaei et al. 2023).

Bar hardening is one of the main concerns associated with protein bars that can be influenced by the aggregation of proteins due to moisture, pH, and temperature changes throughout storage (Zhou et al. 2013). The size and charge of proteins, as well as the composition of protein bars, are other parameters influencing the texture and functionality of the bars. Hence, the selection of raw materials, including protein sources, has a direct impact on the texture of protein bars during the storage period. Imtiaz, Kuhn‐Sherlock, and Campbell (2012) reported that blending different proteins (whey protein concentrate and milk protein concentrate) reduced the hardness of the protein bars. Incorporating protein hydrolysates into the formulation of protein bars is one of the suggested methods to modify the textural changes of the bars. This can be attributed to the plasticizing properties exhibited by the hydrolysates (Jiang et al. 2021). Different studies have indicated that the water retention ability of proteins improves through hydrolysis, resulting from an increase in terminal carboxyl and amino groups (Alahmad et al. 2022; Sinha et al. 2007). This could ultimately impact the characteristics of the bars, including their firmness, viscosity, and protein gel strength (Munir et al. 2016).

The objective of this study is to evaluate the feasibility of incorporating blue whiting fish protein hydrolysate (BWFPH) as a novel protein source in protein bars alongside whey protein isolate (WPI) and pea protein isolate (PPI). Specifically, this study aims to investigate how BWFPH affects the textural, nutritional, and sensory qualities of protein bars and to optimize the formulation for a balanced profile of texture and sensory appeal.

## 2 Materials and Methods

### 2.1 Materials

Whey protein isolate (WPI) with a protein content of 90/100 g and pea protein isolate (PPI) with a protein content of 69 g/100 g were purchased from Myprotein (Northwich, UK). Desalted blue whiting fish protein isolates (BWFPH) with a protein content of 92.6 g/100 g were supplied by Bio‐Marine Ingredients Ireland Ltd. (Castleblayney, Ireland). The physicochemical and nutritional properties of BWFPH used in this research are reported in a previous study by Harnedy‐Rothwell et al. (2021). Pure 100% virgin coconut oil (KTC edibles, Wednesbury, UK), sunflower seed oil (Veraneo, Spain), wheat germ (Odlums Group, Dublin, Ireland), glucose syrup (DGF Service, France), vanilla extract (Blanquefort, France), maltodextrin (Sosa ingredients SL, Barcelona, Spain), and sucralose (Splenda, Heartland Consumer Products LLC, Netherlands) were purchased from a local shop in Galway, Ireland.

### 2.2 Preparation of Protein Bars

The protein paste formulation was according to our preliminary trials (data not shown) and it consisted of 44.5/100 g of protein source, 10/100 g glucose syrup, 10/100 g of oil mixture (coconut oil and sunflower seed oil), 10.5/100 g wheat germ, 2/100 g sucralose, 11/100 g water, 2/100 g maltodextrin, and 10/100 g vanilla extract. The dry ingredients were combined in a mixing bowl. After heating the oil mixture to 55°C, it was combined with dry ingredients and mixed extensively. Then, water and vanilla extract were combined with the mixture, and once the heated glucose syrup was added, the ingredients were thoroughly mixed to create a cohesive dough. Once the dough was molded into bar shapes, they were securely enclosed in polyethylene bags and stored at room temperature for subsequent analysis. The percentage contribution of WPI and PPI in the total protein mixture varied from 0% to 100%, while for BWFPH, it was selected from 0% to 50%. Table 1 presents the compositions of WPI, PPI, and BWFPH mixtures at the different design points.

**TABLE 1:** Composition of different formulations based on experimental design and their nutritional values.

Run	Ingredients (g/100 g of sample)										Nutritional value (%)					
	WPI	PPI	BWFPH	Glucose syrup	Oil mixture	Wheat germ	Sucralose	Water	Maltodextrin	Vanilla extract	AC	TC	MC	FC	PC	EV (kCal)
1	12.79	9.46	22.25	10	10	10.5	2	16	2	5	2.73	24.95	22.13	10.65	39.54	354
2	21.84	18.89	3.76	10	10	10.5	2	16	2	5	2.05	28.91	19.01	10	40.03	366
3	21.99	0.25	22.25	10	10	10.5	2	16	2	5	2.64	25.65	20.2	10.07	41.44	359
4	44.5	0	0	10	10	10.5	2	16	2	5	1.71	30.02	18.06	10.07	40.14	371
5	2.22	42.27	0	10	10	10.5	2	16	2	5	2.13	23.91	21.95	12.84	39.17	368
6	31.39	0	13.10	10	10	10.5	2	16	2	5	2.16	27.95	17.75	10.21	41.93	371
7	2.30	20.13	22.07	10	10	10.5	2	16	2	5	2.9	21.09	23.42	11.04	41.55	350
8	0	30.68	13.82	10	10	10.5	2	16	2	5	2.55	24.15	23.35	11.02	38.93	352
9	31.39	0	13.10	10	10	10.5	2	16	2	5	2.19	25.66	19.31	10.62	42.22	367
10	33.79	10.71	0	10	10	10.5	2	16	2	5	1.7	28.53	19.45	10.68	39.64	369
11	13.47	31.02	0	10	10	10.5	2	16	2	5	1.96	24.77	23.33	11.08	38.86	354
12	10.93	22.14	11.43	10	10	10.5	2	16	2	5	2.38	23.44	22.51	10.84	40.83	355
13	2.22	42.27	0	10	10	10.5	2	16	2	5	2.19	24.54	21.28	12.79	39.17	370
14	0	30.68	13.82	10	10	10.5	2	16	2	5	2.35	22.85	23.16	11.23	40.41	354
15	10.93	22.13	11.43	10	10	10.5	2	16	2	5	2.2	22.42	23.64	11.03	40.71	352
16	21.84	18.89	3.76	10	10	10.5	2	16	2	5	1.83	29.08	18.58	10.63	39.88	372

Abbreviations: AC, ash content; BWFPH, blue whiting fish protein hydrolysate; EV, energy value; FC, fat content; MC, moisture content; PC, protein content; PPI, pea protein isolate; TC, total carbohydrate content; WPI, whey protein isolate.

### 2.3 Proximate Composition

The moisture content of bars (AOAC 934.01), protein content (AOAC 920.87), ash content (AOAC 923.03), and fat (AOAC 922.06) were determined using official standardized methods (AOAC, 2012). The carbohydrate contents and the energy value of the samples were calculated according to the formulas below:

1.
$$\text{Carbohydrates content}\;\left(\%\right)=100-\text{moisture content}\;\left(\%\right)-\text{protein content}\;\left(\%\right)-\mathrm{ash}\;\text{content}\;\left(\%\right)-\text{crude}\;\mathrm{fat}\;\left(\%\right)$$
2.
$$\text{Energy value}\;\left(\frac{\text{kcal}}{100g}\right)=4\;c\text{arbohydrates}\;\left(\%\right)+4\;\text{proteins}\;\left(\%\right)+9\text{crude}\;\mathrm{fat}\;\left(\%\right)$$
3.
$$\text{Energy value}\;\left(\frac{\text{kcal}}{100g}\right)=4\;\text{carbohydrates}\;\left(\%\right)+4\;\text{proteins}\;\left(\%\right)+9\;\text{crude}\;\mathrm{fat}\;\left(\%\right)$$

### 2.4 Color Characterization

The surface color of the bars was analyzed on the first day of storage (day 1) using the CIE L* a* b* color system with a calibrated Chroma Meter CR‐400 (Konica Minolta Inc., Osaka, Japan) following the method suggested by Rezagholi and Hesarinejad (2017). Five random samples from each treatment were selected, and their surface color parameters—L* (Lightness), a* (redness for positive values and greenness for negative values), and b* (yellowness for positive values and blueness for negative values)—were measured and recorded (Khodaei, Hamidi‐Esfahani, and Rahmati 2021).

### 2.5 Textural Study

Texture measurements were carried out on days 1, 14, and 28 of storage at ambient temperature using TA.XT2 Texture Analyzer (Stable Micro Systems, Surrey, England) equipped with a 5 kg load cell. The cutting force (N) was recorded as the maximum force needed to cut protein bars (1.5 × 2 cm) using a cutter probe (speed of 2 mm/s). For the firmness analysis, protein bars (2 × 2 cm) were subjected to a compression test using a 50 mm cylinder probe. The maximum force required to compress the bars to 50% strain was measured and reported in Newtons (N). The probe speed was set to 1 mm/s, with a strain level of 50% (Khodaei et al. 2023).

### 2.6 Sensory Evaluation

For the sensory evaluation, a total of 15 semi‐trained panelists were selected. The chosen individuals ranged in age from 18 to 55 and consisted of nine females and six males. The participants were recruited from the students and academic staff in the food and culinary school (Atlantic Technological University, Galway) who had some previous background in sensory analysis. The sample group was selected among individuals who have prior knowledge and experience with protein bars and are inclined to consume high‐protein bars. Additionally, participants were screened for any medical or dietary restrictions. The possible participants were all asked about possible food allergies to wheat, milk proteins, fish, and seafood. The study was reviewed and approved by the research ethics committee (IREC) of the Atlantic Technological University of Galway. Afterward, the participants were instructed on the objectives of the study, and they were asked to read and sign the consent form. The sensory assessment was conducted within the sensory analysis laboratory, where participants were seated in individual booths. Each participant had access to a tablet device equipped with Compusense software (Compusense Inc., Guelph, Ontario, Canada). The participants were trained to evaluate each sample for aroma, taste, and overall liking. This evaluation was conducted using a standardized 9‐point hedonic scale where a score of 1 indicated highly disliked, while a score of 9 represented highly liked (Khodaei et al. 2023).

### 2.7 Statistical Analysis

A simplex centroid mixture design approach was used to identify the best combinations of WPI, PPI, and BWFPH as independent factors and nutritional content, texture, color, and sensory characteristics as dependent variables (Table 1). Various models, including linear, quadratic, and cubic models, were employed to evaluate the effects of protein combinations on the responses. The selected model for each response was determined based on a *p‐*value of less than the significance level (*p* ≤ 0.05) and lack of fit of *p* > 0.05. The estimated regression coefficient values for the responses are presented in Table 3. The experimental design and statistical analysis (one‐way ANOVA) were conducted using Design‐Expert software (Version 12, StatEase, Minneapolis, USA). The sensory data were analyzed using Compusense software (Compusense Inc., Guelph, Ontario, Canada).

## 3 Results and Discussion

### 3.1 Proximate Composition

**FIGURE 1:**
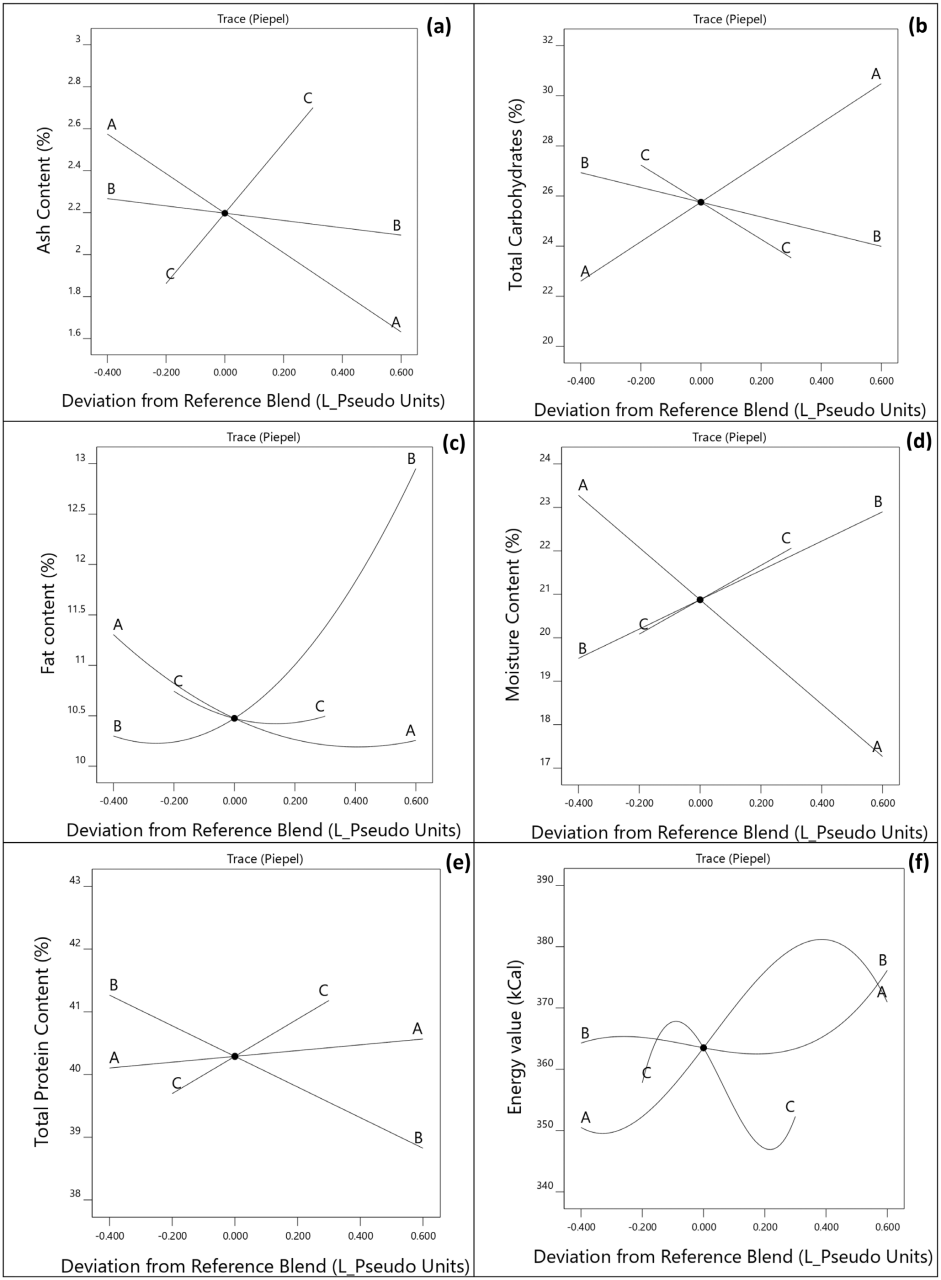
Cox response trace plot for (a) Ash content. (b) Total carbohydrates. (c) Fat content. (d) Moisture content. (e) Total protein content. (f) Energy value of protein bars in relation to different combinations of WPI (A), PPI (B), and BWFPH (C).

Table 1 presents the proximate composition (ash content, total carbohydrates, total fat, total protein, and moisture content) of the bars formulated with various combinations of WPI, PPI, and BWFPH. As shown in Figure 1a, the relationship between ash content and protein concentration was linear, ranging from 1.7% to 2.9%. The Cox trace plot figure also illustrates that the ash content of the protein bars significantly increased with the addition of BWFPH, while it decreased with the inclusion of WPI and PPI. This finding can be attributed to the higher mineral content in BWFPH (5.93%), compared to WPI (1.84%) and PPI (4.1%). The total carbohydrate content of the protein bars ranged from 21.09% for bars containing the highest amount of BWFPH to 30.02% for the bars containing the highest amount of WPI. The Cox trace plot revealed a positive correlation between the concentration of WPI and the total carbohydrate content in the bars. However, the correlation was negative for PPI and BWFPH. It has been reported that consumers prefer protein bars labeled as having “low carbohydrate content” (Harwood and Drake 2019). As a result, there is an increased desirability for the development of protein bars with higher levels of protein and reduced carbohydrate content. The fat content of the bars ranged from 10% w/w for the samples containing higher amounts of WPI to 12.8% for the samples containing higher amounts of PPI. This difference in fat content could be attributed to the varying oil‐binding capacities of the proteins. Table 3 indicates a quadratic relationship between fat content and protein combinations, with the interaction between the WPI and PPI significantly reducing the fat content of the bars (*p* ≤ 0.05). The protein content of bars ranged from 38.86% to 42.22%, with the lowest protein content observed in samples containing a higher proportion of PPI. This variation in protein content among samples can be attributed to the differing protein concentrations in each source: WPI, PPI, and BWFPH containing 90, 80, and 92.6/100 g of protein, respectively. The Cox trace plot (Figure 1e) indicated a linear correlation between the concentration of BWFPH and the protein content in the bars. Similar to our results, Maleki et al. (2023) found that nutrition bars containing 30% of Cowpea protein had higher protein content and lower fat compared to other samples. Jabeen et al. (2022) also stated that incorporating 10% roasted chickpea flour into protein bars elevated the protein content and profile. The moisture content of the bars ranged from 17.75% to 23.64%, which is typical for intermediate moisture‐content foods like high‐protein bars (Samuel and Peerkhan 2020). The moisture content of our protein bars was similar to that of cowpea protein‐containing bars reported by Maleki et al. (2023), which fall into the intermediate moisture food category (10%–40% moisture). Figure 1d illustrates that protein bars with higher amounts of BWFPH and PPI had the highest moisture content, while those with higher amounts of WPI had lower moisture content. This variation in moisture content among the protein sources can be attributed to the differences in their water activity and water‐holding capacity. BWFPH, with its high solubility, has a greater water‐binding capacity compared to WPI and PPI. Moisture content directly affects both the texture and shelf‐life of protein bars. Our findings are consistent with those of Banach et al. (2016), who observed that high‐protein bars (containing 30% w/w protein) made with milk protein concentrate (MPC), micellar casein concentrate, and reduced calcium MPC had moisture content ranging from 16.1% to 17.5%. The energy value of the bars in our study ranged from 350 to 372 kcal per 100 g. This is notably lower than the energy value of approximately 400 kCal reported by Małecki, Tomasevic, and Sołowiej (2022) for WPC‐glucose syrup‐based bars. However, the energy values of our protein bars were comparable to those of whey‐millet protein bars developed by Samuel and Peerkhan (2020), which ranged from 332 to 379 kcal per 100 g. Similarly, Zhou et al. (2022) reported energy values for hemp protein— *Tenebrio molitor* larvae protein bars ranging from 336.98 to 56.13 kcal, aligning closely with the findings of our research. The contour plots for the energy values indicate that the bars containing WPI and PPI had the highest energy value, while the addition of BWFPH decreased the energy value of the final bars (Figure S1f). BWFPH‐containing bars in our study demonstrated a higher protein and carbohydrate content compared to the findings of previous studies by Szydłowska et al. (2020) and Malecki et al. (2020) along with a reduced fat content. This suggests that the incorporation of BWFPH into the bars not only enhances the protein content relative to other protein sources but also reduces the reliance on oils for shaping and textural properties due to its higher moisture content. Consequently, this results in bars with elevated protein content and reduced fat content.

### 3.2 Texture Hardness

**FIGURE 2:**
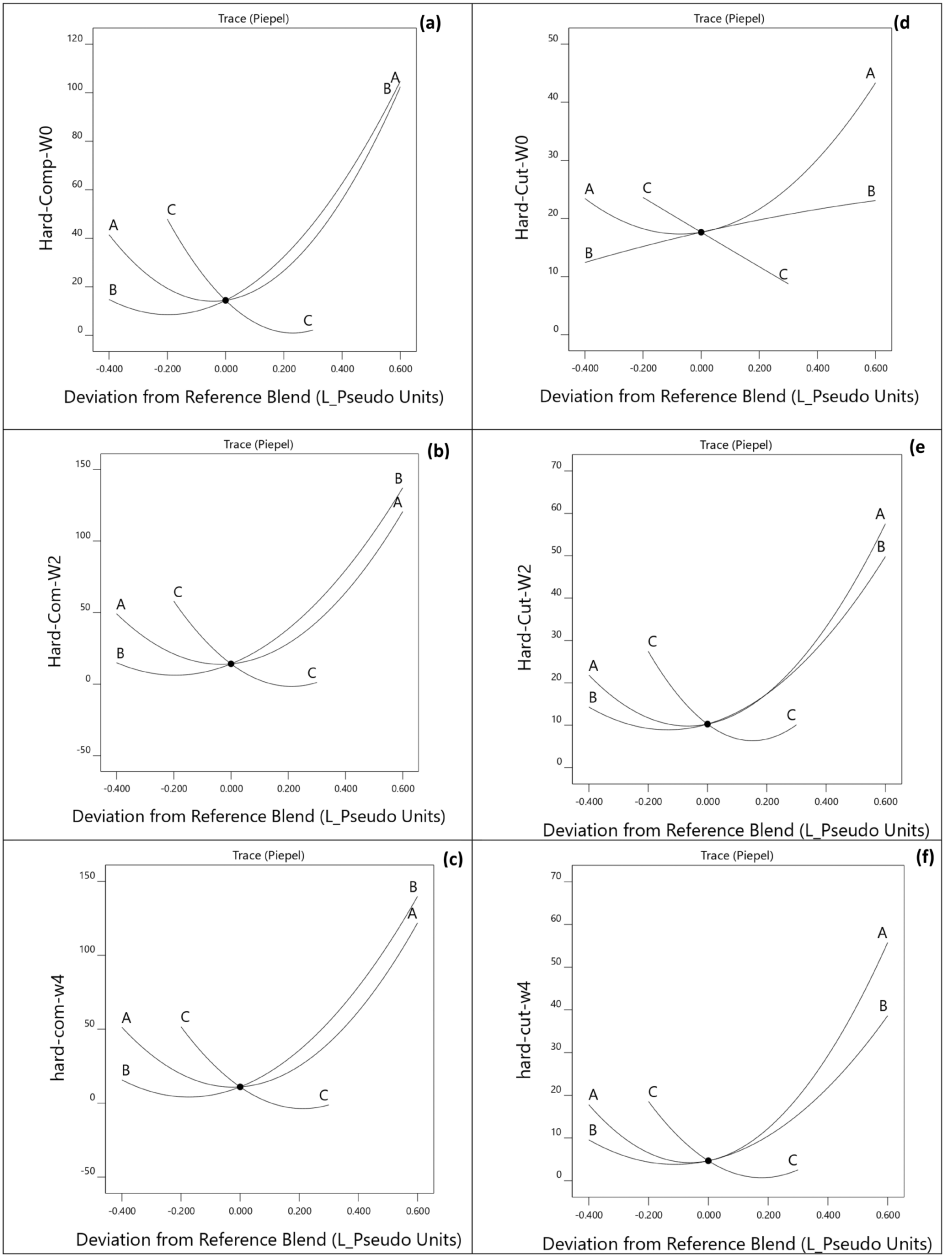
Cox response trace plot for Firmness value (N) at the day 1 (a), day 14 (b), day 28 (c) of storage and cutting force value (N) in protein bars at the day 1 (d), day 14 (e), and day 28 (f) of storage in relation to different combination of WPI (A), PPI (B), and BWFPH (C).

Table 2 presents the firmness values of the protein bars at days 1, 14, and 28 of storage. On day 1, the firmness values ranged from 5.93 ± 1.7 N for bars containing 50:50 BWFPH and WPI to 100.33 ± 8.6 N for bars utilizing 100% WPI as the protein source. Bars formulated with 95% PPI did not maintain an acceptable texture, which can be attributed to the weaker protein network and reduced water absorption capacity of PPI. Therefore, the texture characteristics, color, and sensory parameters of these bars were not assessed in this study. Evaluation of the firmness of the protein bars during the storage period revealed a gradual increase when stored at room temperature. For instance, the firmness of bars formulated with 100% WPI as the protein source exhibited a gradual increase over the storage period. The firmness values for these bars were 100.33 ± 8.6 N on day 0, 120.3 ± 23.2 N on day 14, and 127.17 ± 6.6 N on day 28. These results confirm the progressive hardening of the protein bars during storage. However, the inclusion of BWFPH significantly slowed the rate of hardening over the storage period. The firmness values for the bars containing a 50:50 blend of BWFPH and WPI remained relatively stable throughout the storage period, with values of 5.93 ± 1.7 on day 1, 4.09 ± 1.3 on day 14, and 4.39 ± 1.5 N on day 28. As illustrated in the Cox response trace plot (Figure 2a–c) and detailed in Table 3, a quadratic relationship between the protein combination and the firmness of protein bars was observed during the storage period. Specifically, the inclusion of both WPI and PPI resulted in increased bar firmness, whereas the addition of BWFPH led to a reduction in firmness value. Bar hardness over time is suggested to be linked to the migration of water from the protein matrix to the glucose and glycerol components, accompanied by the structural separation of aggregated protein (Loveday et al. 2010). BWFPH has high water solubility (~80%) and a low molecular weight (Egerton et al. 2018), which may influence the rheology and texture of bars containing BWFPH by reducing the rate of bar hardening. Consistent with our findings, McMahon, Adams, and McManus (2009) reported that the firmness of WPI‐based protein bars increased over time. However, protein bars containing partially hydrolyzed WPI remained softer compared to those made with unhydrolyzed WPI. Similarly, Bekiroglu et al. (2024) reported that the incorporation of whey protein hydrolysates significantly reduced the hardness of muffins. Wang et al. (2023) also observed an increase in the hardness of WPI‐ and casein‐based protein bars during the storage period, attributing this phenomenon to disulfide cross‐linking and glycosylation reactions. WPI is particularly susceptible to glycosylation, which can lead to the formation of protein aggregates. This is important because bars hardening during storage presents a major challenge in the production of protein bars, and the incorporation of protein hydrolysates has been shown to mitigate the rate of hardness development, resulting in a softer and more flexible texture in high‐protein bars. Sugar crystallization, water migration within the product, protein self‐aggregation, phase separation, and Maillard reactions have been identified as key factors contributing to the hardening of bars during storage (Jiang et al. 2021).

**TABLE 2:** Textural, color, and sensorial properties of protein bars from different formulations.

Run	Textural properties (*N*)						Color parameters			Sensorial attributes		
	F1	F14	F28	C1	C14	C28	L*	a*	b*	Aroma	Taste	Overall liking
1	7.68 ± 1.7	4.08 ± 1.3	4.39 ± 1.5	6.08 ± 0.6	13.93 ± 3.4	5.96 ± 0.4	59.82 ± 3.3	7.16 ± 0.1	33.01 ± 1.0	5.17 ± 1.4	4 ± 1.8	4.5 ± 1.7
2	38.13 ± 5.4	40.89 ± 9.4	40.18 ± 8.8	17.83 ± 1.4	28.14 ± 5.9	23.00 ± 4.5	57.62 ± 1.4	8.49 ± 0.2	28.85 ± 0.3	5.71 ± 0.9	5 ± 1.1	5.29 ± 1.2
3	5.93 ± 1.7	7.2 ± 2.4	4.77 ± 0.8	5.76 ± 0.3	11.79 ± 1.2	5.36 ± 0.5	59.61 ± 2.3	5.79 ± 0.3	33.54 ± 0.8	4.83 ± 1.3	3.33 ± 1.6	3.8 ± 1.5
4	100.33 ± 8.6	120.3 ± 23.2	127.16 ± 6.6	44.6 ± 4.4	60.52 ± 5.0	61.34 ± 7.1	53.17 ± 1.2	6.27 ± 0.4	25.97 ± 0.7	5.86 ± 1.4	6 ± 1.9	5.14 ± 1.7
5	NS	NS	NS	NS	NS	NS	NS	NS	NS	NS	NS	NS
6	17.00 ± 2.1	16.69 ± 6.2	18.09 ± 1.3	11.04 ± 0.8	14.35 ± 6.8	9.73 ± 0.8	54.19 ± 2.1	6.13 ± 0.2	30.04 ± 1.1	4.57 ± 1.2	4.71 ± 1.5	6 ± 0.8
7	8.66 ± 1.8	13.79 ± 2.4	13.22 ± 2.4	15.06 ± 7.5	13.40 ± 2.0	6.66 ± 0.6	59.45 ± 0.7	8.06 ± 0.4	31.73 ± 0.5	4 ± 1.6	2.43 ± 1.7	2.57 ± 1.6
8	51.60 ± 6.5	62.07 ± 7.2	66.11 ± 11.4	23.17 ± 1.9	24.42 ± 5.7	21.55 ± 4.0	60.7 ± 0.6	7.78 ± 0.1	30.35 ± 0.2	3.5 ± 1.4	3.33 ± 0.9	3.5 ± 0.8
9	16.09 ± 6.0	17.55 ± 4.2	15.92 ± 6.7	15.29 ± 9.3	14.94 ± 4.4	8.22 ± 0.6	54.04 ± 1.3	6.63 ± 0.3	30.83 ± 1.0	4 ± 0.7	6 ± 1.7	4.43 ± 1.7
10	73.65 ± 8.7	81.48 ± 10.5	66.45 ± 4.7	34.18 ± 1.2	33.71 ± 2.5	22.98 ± 2.4	54.05 ± 1.3	7.65 ± 0.3	26.28 ± 1.0	5.14 ± 1.2	6 ± 1.9	5.86 ± 1.4
11	51.83 ± 8.2	68.04 ± 7.1	64.56 ± 6.4	21.56 ± 0.8	29.32 ± 3.9	17.60 ± 1.1	58.87 ± 1.1	8.37 ± 0.2	27.60 ± 0.6	4.67 ± 0.6	4 ± 1.8	3 ± 0.8
12	18.90 ± 7.9	21.91 ± 5.9	21.26 ± 1.5	24.26 ± 4.0	13.19 ± 0.8	6.59 ± 0.3	58.47 ± 1.8	8.05 ± 0.3	29.61 ± 1.2	4.57 ± 1.2	4 ± 1.2	4.57 ± 1.3
13	NS	NS	NS	NS	NS	NS	NS	NS	NS	NS	NS	NS
14	46.83 ± 9.1	50.51 ± 10.3	47.24 ± 4.4	22.15 ± 1.5	21.52 ± 2.4	16.56 ± 1.7	60.13 ± 1.6	7.75 ± 0.1	30.17 ± 0.5	3.5 ± 0.9	3 ± 1.8	3 ± 1.1
15	16.29 ± 1.4	18.81 ± 3.5	17.75 ± 2.4	21.97 ± 2.7	10.74 ± 0.6	6.00 ± 0.1	59.51 ± 0.4	8.11 ± 0.1	29.85 ± 0.2	4 ± 1.0	4 ± 1.9	4 ± 1.4
16	16.96 ± 4.5	22.53 ± 4.2	19.54 ± 3.6	16.42 ± 1.2	9.8267 ± 2.3	7.49 ± 0.3	57.54 ± 1.4	8.26 ± 0.2	28.18 ± 0.8	5 ± 1.4	5.29 ± 2.2	6 ± 1.6

*Note:* Values are expressed as mean ± standard deviation (*n* = 3).

Abbreviations: C1, cutting force day 1; C14, cutting force day 14; C28, cutting force day 28; F1, firmness day 1; F14, firmness day 14; F28, firmness day 28; NS, data not included.

**TABLE 3:** Estimated regression coefficients for the responses (in component proportions).

Term	Nutritional value (%)						Textural properties (*N*)						Color parameters			Sensorial attributes		
	AC	TC	MC	FC	PC	EV (kCal)	F1	F14	F28	C1	C14	C28	L*	a*	b*	Aroma	Taste	Overall liking
WPI	1.63	30.48	17.27	10.26	40.57	370.9	102.4	120.6	121.8	43.3	57.6	55.7	47.1	9.2	24.4	5.5	6.6	5.3
PPI	2.09	23.99	22.90	12.95	38.82	376.1	104.7	137.2	139.8	23.1	49.8	38.6	64.0	6.2	29.4	3.1	3.7	−0.4
BWFPH	3.54	19.83	24.04	11.67	42.67	860.3	82.9	120.2	108.8	−5.8	78.1	48.9	90.6	10.2	54.2	8.4	1.7	−4.9
WPI × PPI				−3.44^a^		−62.98	−222.7^a^	−283.6	−316.6^a^	−38.4	−104.9^a^	−114.6	3.8	8.2^a^	−0.2	4.5		9.6^a^
WPI × BWFPH				−1.92		−1024.7	−365.9^a^	−474.4^a^	−485.2^a^	−65.4	−225.5^a^	−197.5^a^	−43.7^a^	−6.4	−17.3	−8.9		14.6
PPI × BWFPH				−5.48		−1063.1	−251.9	−370.3	−351.9^a^	44.9	−168.3^a^	−109.1^a^	−56.5^a^	1.3	−37.9^a^	−6.2		21.9^a^
WPI × PPI × BWFPH						1354.9												
Standard deviation	0.10	1.40	1.20	0.36	0.78	10.35	9.97	9.16	9.93	4.35	5.59	6.52	1.25	0.23	0.4	0.45	0.50	0.65

Abbreviations: AC, ash content; C1, cutting force day 1; C14, cutting force day 14; C28, cutting force day 28; EV, energy value; F1, firmness day 1; F14, firmness day 14; F28, firmness day 28; FC, fat content; MC, moisture content; PC, protein content; TC, total carbohydrate content.

^a^ The interaction between terms are significantly different at *p* ≤ 0.05.

The cutting force value of the bars was assessed throughout the storage period (Figure 2d–f and Figure S2d–f). The force required to cut the bars ranged from 5.76 ± 0.3 N for samples containing 50:50 blend of BWFPH and WPI to 44.6 ± 4.4 N for samples formulated with 100% WPI (Table 2). The data indicate that the cutting force value increased over the storage period. For example, for bars containing 100% WPI, the cutting force values at days 1, 14, and 28 were 44.6 ± 4.4, 60.52 ± 5.0, and 61.34 ± N, respectively. On the contrary, samples formulated with a 50:50 blend of BWFPH and WPI exhibited the lowest cutting force values during the storage period. The cutting force values for these bars were 5.76 ± 0.3, 11.79 ± 1.2, and 5.36 ± 0.5 N at days 1, 14, and 28, respectively. The quadratic model also confirmed that the interaction between WPI and PPI increased the hardness value of the bars, while the addition of BWFPH reduced it (Table 3). This phenomenon is associated with the water binding and plasticizing properties of protein hydrolysates, which help to reduce protein aggregation and interact with other ingredients in bar formulations, such as carbohydrates and fats. These properties play a significant role in reducing the hardness of protein bars during the storage period.

### 3.3 Color Characteristics

**FIGURE 3:**
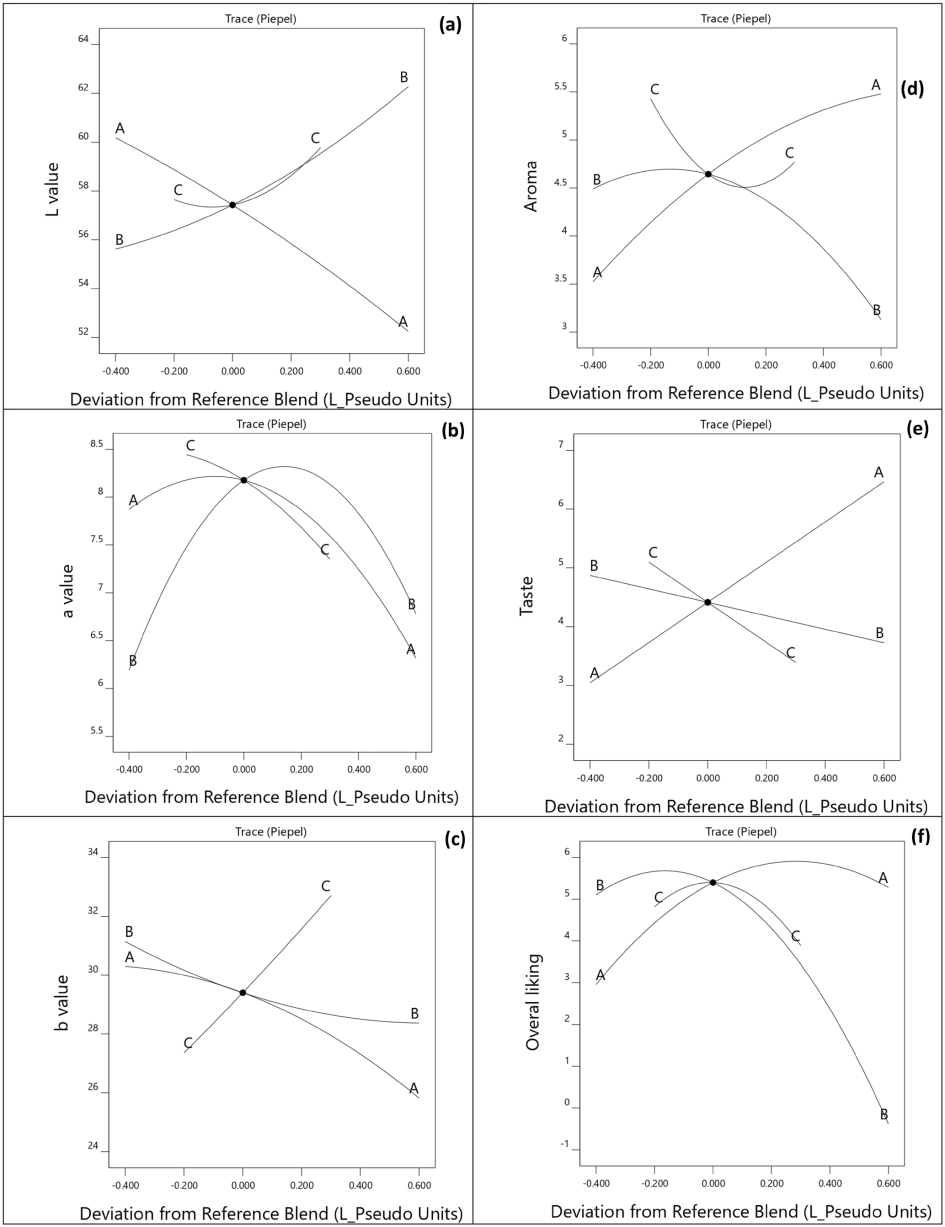
Cox response trace plot for colour parameters: L* (a), a*(b), b* (c) and the sensorial parameters: aroma (d), taste (e), and overall liking (f) in protein bars in relation to different combination of WPI (A), PPI (B), and BWFPH (C).

The color of protein bars plays a crucial role in consumer perception and is significantly influenced by the selection of ingredients used in their formulation (Malecki et al. 2020). Table 2 presents the color parameters (L*, a*, and b*) for bars formulated with various combinations of WPI, PPI, and BWFPH. The lightness (L*) values of the protein bars ranged from 53.17 ± 1.2 to 60.7 ± 0.6. The lowest lightness was observed in samples with higher concentrations of WPI, while bars containing PPI demonstrated the highest L* values. The higher brightness observed in PPI‐containing bars may indicate a slower fat‐binding ability compared to those containing WPI and BWFPH (Maleki et al. 2023). Other studies have also shown the effect of protein sources on the lightness of bars. For example, Khalil et al. (2024) reported that adding cricket flour significantly reduced the lightness of milk protein‐based protein bars. As shown in Table 3, the estimated regression coefficients reveal that the interaction between BWFPH with PPI and WPI was statistically significant (*p* ≤ 0.05). The Cox trace plot (Figure 3a) demonstrates that the lightness of the bars increased with the addition of PPI and BWFPH, while the inclusion of WPI led to decrease in lightness.

The a* values of the protein bars ranged from 5.80 ± 0.3 to 8.49 ± 0.2 with the highest value observed in bars containing a combination of all three protein sources. This increase in the a* values may be attributed to potential reactions between the proteins and change in the color of the bars. The Cox trace plot for a* (Figure 3b) indicated that the addition of BWFPH and WPI decreased the redness of the bars. The estimated regression coefficient in Table 3 show that the interactions between WPI and PPI had a significant effect on the a* value. Specifically, blending WPI and PPI resulted in a significant increase in red color intensity of the bars, which was mitigated when BWFPH was included in the formulation.

The b* values of the protein bars, reflecting their yellowness, as influenced by combinations of WPI, PPI, and BWFPH, are presented in Figure 3c. The yellowness (b*) ranged from 25.97 ± 0.7 to 33.54 ± 0.8, with an increase in b* observed upon the addition of BWFPH to the formulation. The lowest yellowness index was observed in bars containing 100% WPI as the sole protein source. As shown in Table 3, a statistically significant quadratic interaction was observed between PPI and BWFPH (*p* ≤ 0.05). BWFPH and PPI powders, which exhibit an amber hue, contrast with the white appearance of WPI powders, likely contributing to the increased yellowness in bars formulated with PPI and BWFPH. The yellow/amber color could enhance the visual appeal of high‐protein products, as studies have shown that human color perception prioritizes blue, yellow, and red as the most rapidly processed hues (Borges et al. 2019). McMahon, Adams, and McManus (2009) also reported that the color parameters of WPI‐based bars were affected by the inclusion of partially hydrolyzed WPI.

### 3.4 Sensory Evaluation

Consumer acceptance of and flavor preference of protein bars can be significantly affected by the choice of protein source. Consequently, blending different proteins or incorporating hydrolysates is recommended to enhance the flavor, texture, and stability of bars (Childs, Yates, and Drake 2007). Table 2 summarizes the sensory evaluation of protein bars made with various combinations of WPI, PPI, and BWFPH. The aroma values of the bars ranged from 3.5 ± 1.4 for bars with PPI: BWFPH in a ratio of 69:31 to 5.86 ± 1.4 for bars containing WPI: PPI in a ratio of 76:24. Analysis of the Cox trace plot indicates that consumers preferred the aroma of bars containing WPI, with a noticeable reduction in aroma preference when WPI was replaced by BWFPH. On the contrary, bars made predominantly with PPI had the lowest aroma acceptance (Figure 3d). As shown in Table 2 and Figure 3e, consumers preferred the taste of bars with higher WPI content, while the inclusion of BWFPH reduced taste acceptance. This aligns with findings by Lu and Zhou (2019), who reported that protein hydrolysates can impart undesirable bitter flavor and a sticky mouthfeel. Tork et al. (2022) also reported that the inclusion of microalgae reduced the sensory evaluation score of dragée snacks. Bars with a higher proportion of WPI received the highest taste ratings. Overall, Table 2 and Figure 3f illustrate that bars with increased levels of WPI had the highest overall preference, consistent with other sensory attributes. However, the inclusion of PPI was associated with a decline in overall preference. The Cox trace plot further confirms that incorporating higher amounts of WPI enhanced the total acceptance, while the addition of PPI and BWFPH led to decreased overall acceptance (Figure 3f).

These findings highlight the crucial role of protein type and concentration in shaping the sensory attributes of high‐protein bars. These results suggest that bars with a higher concentration of WPI are more preferred by consumers. This preference is consistent with the widespread use of milk‐based proteins in commercial protein bars, known for their desirable technological and sensory properties. WPI‐based protein bars, in particular, are preferred due to their firmer texture and superior textural properties, which can be attributed to the water‐holding and gelling capacity of WPI, leading to enhanced sensory acceptance. On the other hand, the inclusion of PPI and BWFPH was associated with decreased consumer acceptance, which may limit their use in formulations. These findings highlight the importance of carefully selecting the type and concentration of proteins when formulating high‐protein bars. When developing new formulation that incorporate novel protein sources, it is essential to consider both the physical and sensory characteristics of the bars to ensure consumer acceptance.

### 3.5 Validation of Model

To optimize the formulation of protein bars, we utilized the mixture response optimization module using Design Expert software. The goal of this study was to develop protein bars with a high BWFPH content while maintaining textural and sensory characteristics comparable to those of commercially available protein bars. Similar to the results of this study, Abedelmaksoud et al. (2021) reported a quadratic model to describe the relationship of firmness and overall sensory acceptance with product composition in Sunroot snack bars. In our study, BWFPH content, textural parameters, and overall acceptance were prioritized in the optimization process. The results indicated that the optimal protein combination for achieving desirable texture and sensory attributes was 76.02% WPI, 6.72% PPI, and 17.26% BWFPH (Figure S4). The predicted outcomes for this formulation included a firmness of 37 N, a cutting force of 23.67 N, and an overall liking score of 5.78. To validate the model, protein bars were prepared using an optimized formulation. The mean observed values for firmness, cutting force, and overall liking of the optimized bars were 46.53 ± 10.58, 15.42 ± 2.66, and 4.27 ± 1.86, respectively. These results suggest that the proposed model is sufficiently accurate for producing protein bars containing BWFPH with acceptable sensory and physical properties. Other researchers also successfully used simplex centroid design method to optimize energy/fiber‐rich bars (Bourekoua et al. 2023) or dairy protein blend bars (Imtiaz, Kuhn‐Sherlock, and Campbell 2012).

## 4 Conclusion

The results from this study showed that the addition of BWFPH increased the protein content of protein bars and improved textural properties by reducing the firmness and bar‐hardening during the storage period. The sensory analysis revealed that WPI‐based protein bars exhibited the highest sensory acceptance, while replacing WPI with PPI and BWFPH reduced the sensory parameters. An optimization process was carried out to achieve a bar containing a high amount of BWFPH and acceptable sensory properties, without compromising the textural and nutritional properties. The optimization process revealed that protein bars containing 76.02% WPI, 6.72% PPI, and 17.26% BWFPH exhibited the highest sensory acceptance and texture characteristics. These findings indicate that the optimized bars formulated with BWFPH can be considered functional high‐protein bars with lower bar hardening compared to protein bars made from a single source of protein such as WPI or PPI, while also exhibiting acceptable sensory properties. Additional research is needed to investigate the functional properties and microbial stability of protein bars containing protein hydrolysates.

## Author Contributions

**Diako Khodaei:** conceptualization (equal), data curation (equal), formal analysis (equal), funding acquisition (equal), investigation (equal), methodology (equal), validation (equal), writing – original draft (equal), writing – review and editing (equal). **Francesco Noci:** conceptualization (equal), data curation (equal), funding acquisition (equal), investigation (equal), methodology (equal), software (equal), supervision (equal). **Lisa Ryan:** investigation (equal), methodology (equal), project administration (equal), resources (equal), supervision (equal), visualization (equal).

## Ethics Statement

This study does not involve any human or animal testing.

## Consent

Written informed consent was obtained from all study participants.

## Conflicts of Interest

The authors declare no conflicts of interest.

## Supporting information


Data S1.

## Data Availability

The data that support the findings of this study are available from the corresponding author upon request.
