# Supplementary material for: Optimizing Protein Bars With Whey Protein Isolate, Pea Protein Isolate, and Blue Whiting ( Micromesistius poutassou ) Fish Protein Hydrolysate: A Simplex‐Centroid Mixture Design Study
Source: Food Sci Nutr. 2024 Dec 19;13(1):e4701. doi: 10.1002/fsn3.4701 (PMC11717050; doi:10.1002/fsn3.4701)
Supplement: Supplementary file 1 — Data S1. [file FSN3-13-e4701-s001.docx]

| 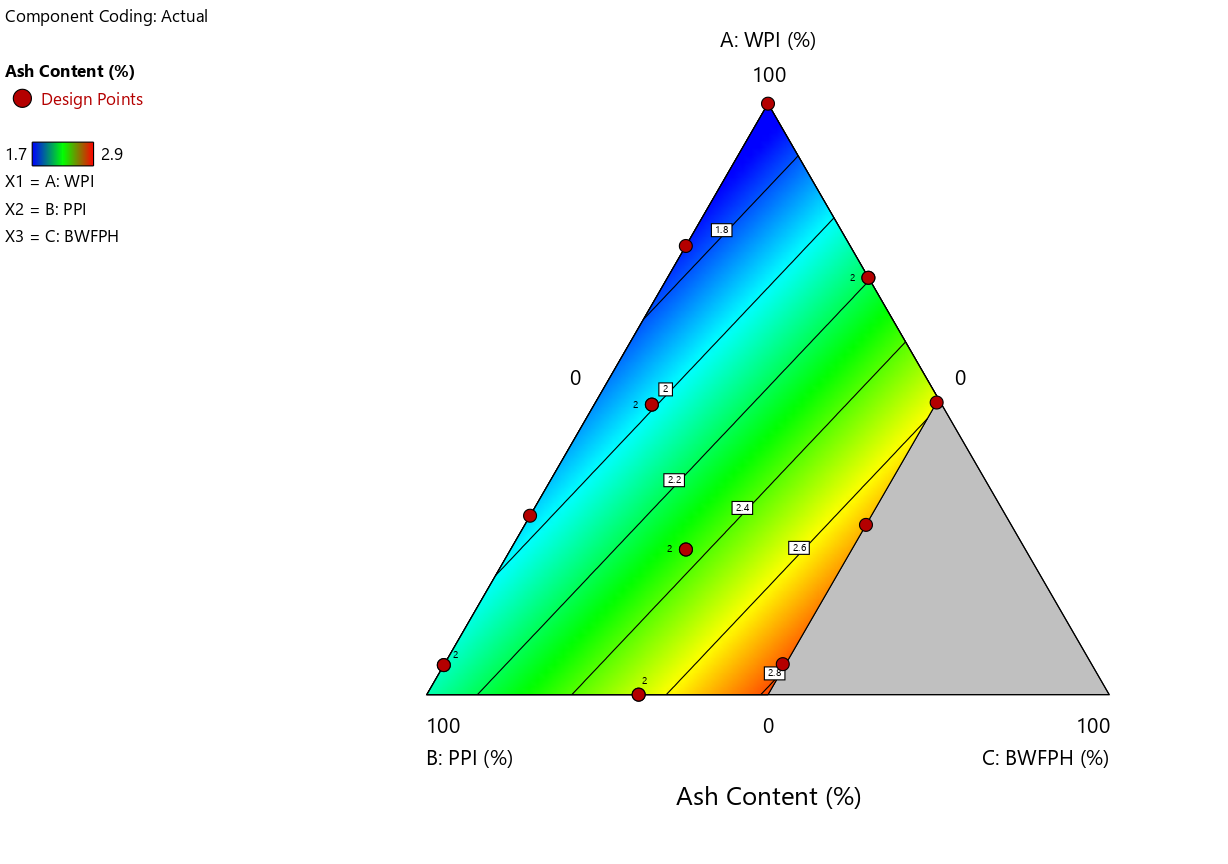  (a) | 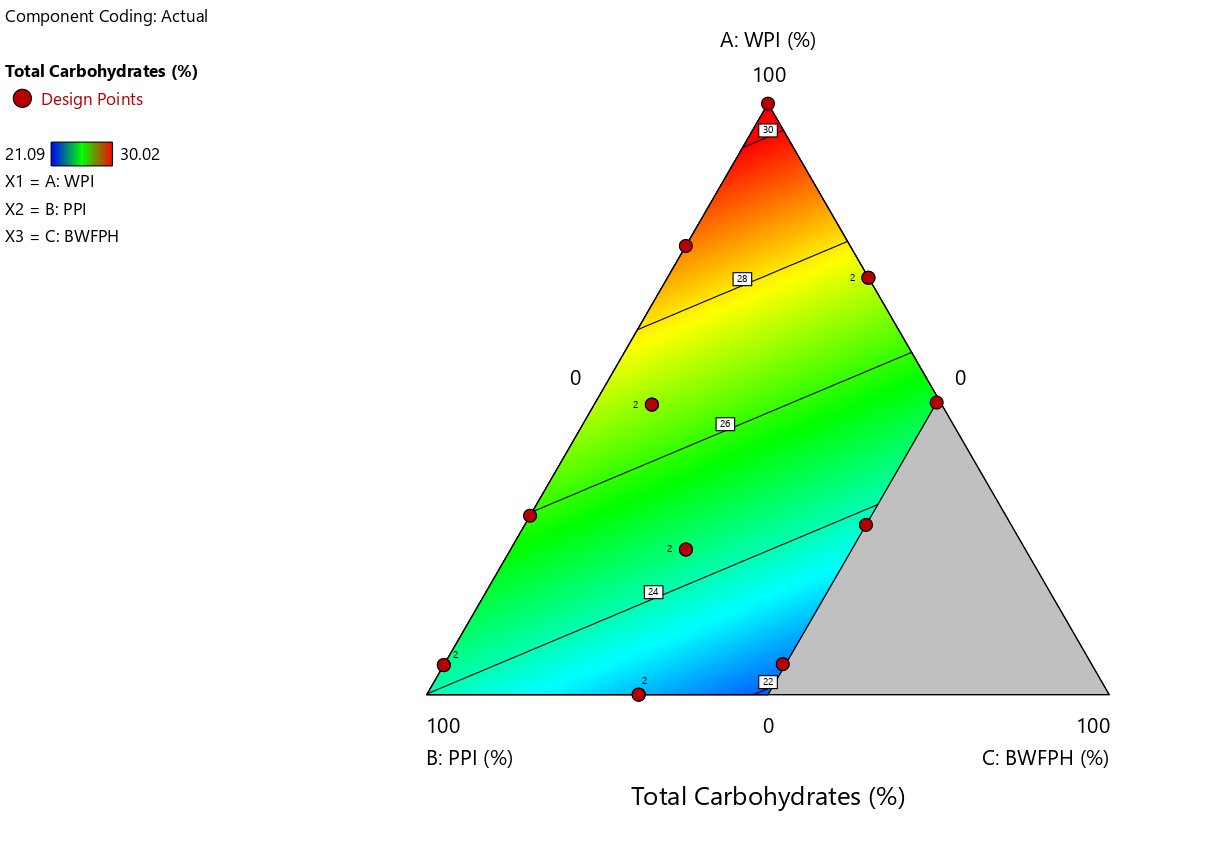  (b) |
| --- | --- |
| 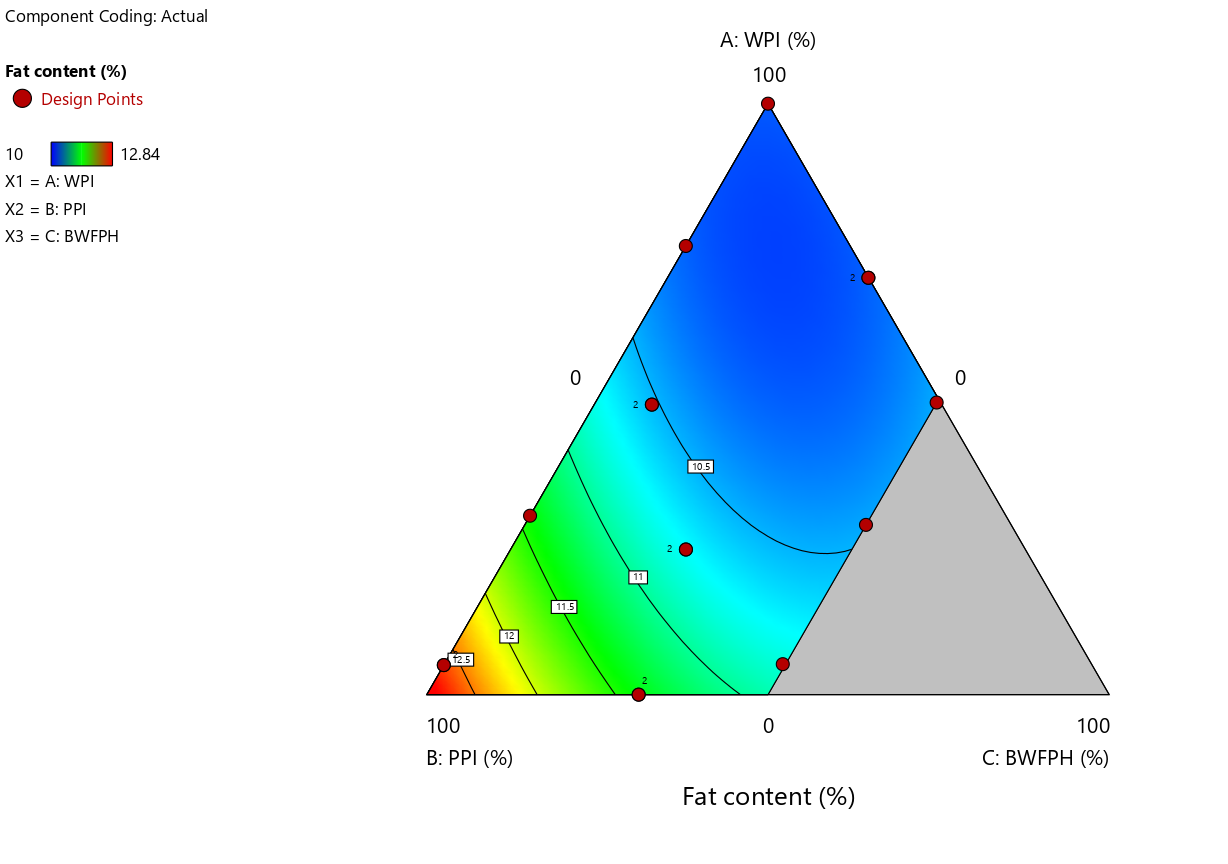  (c) | 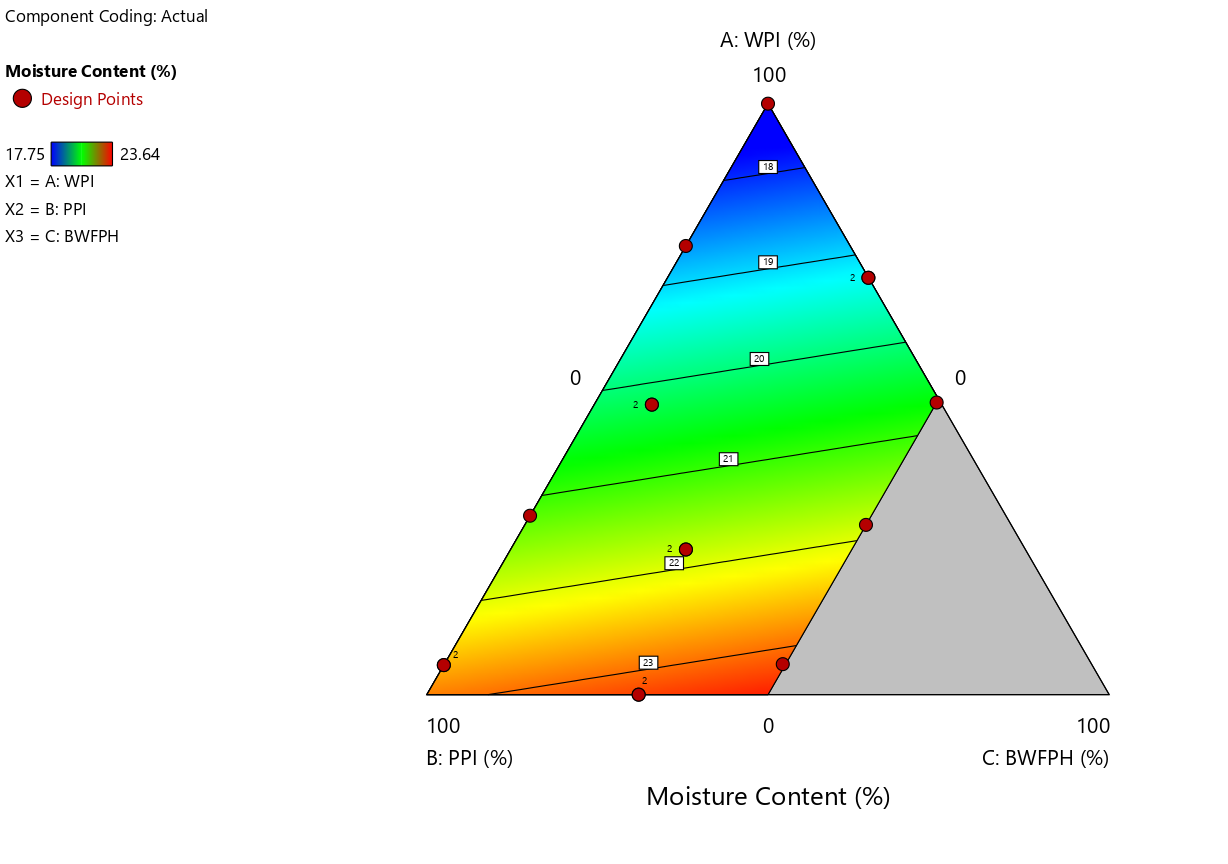  (d) |
| 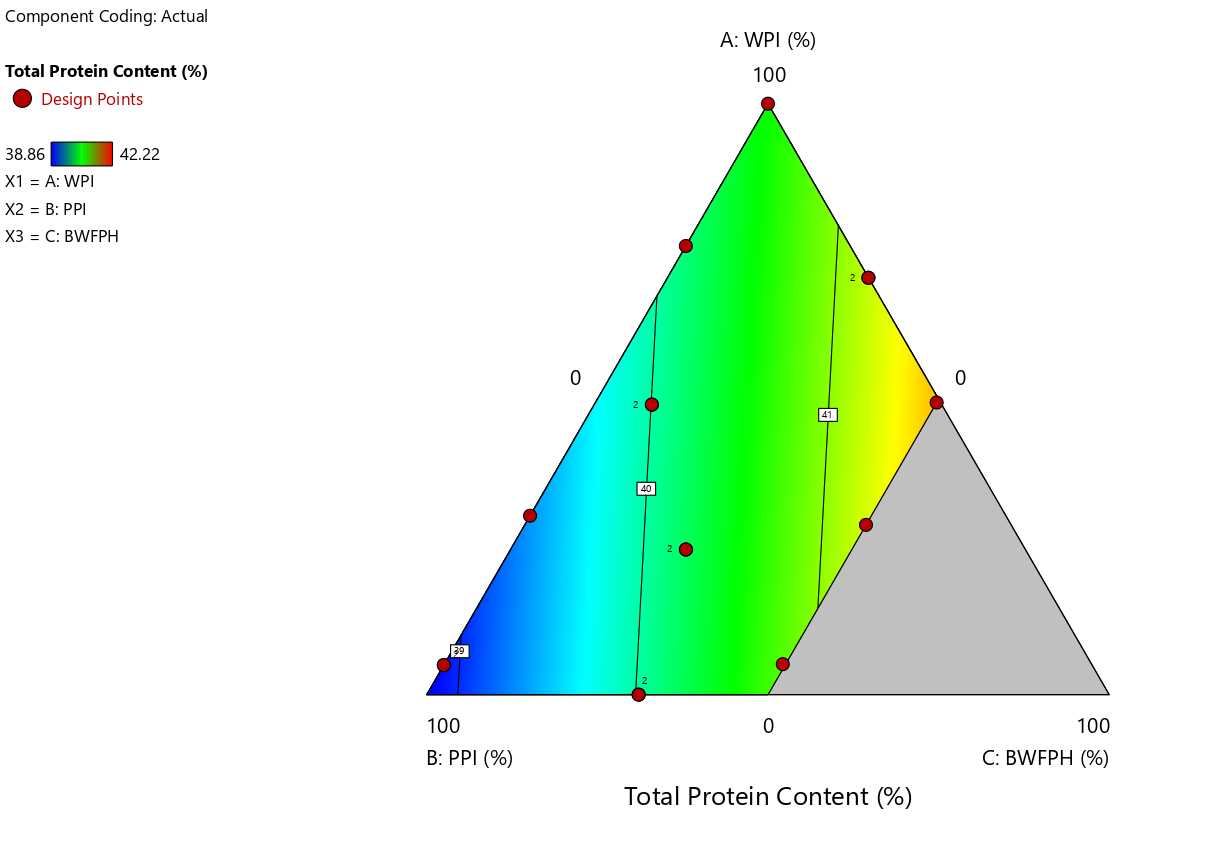  (e)] | 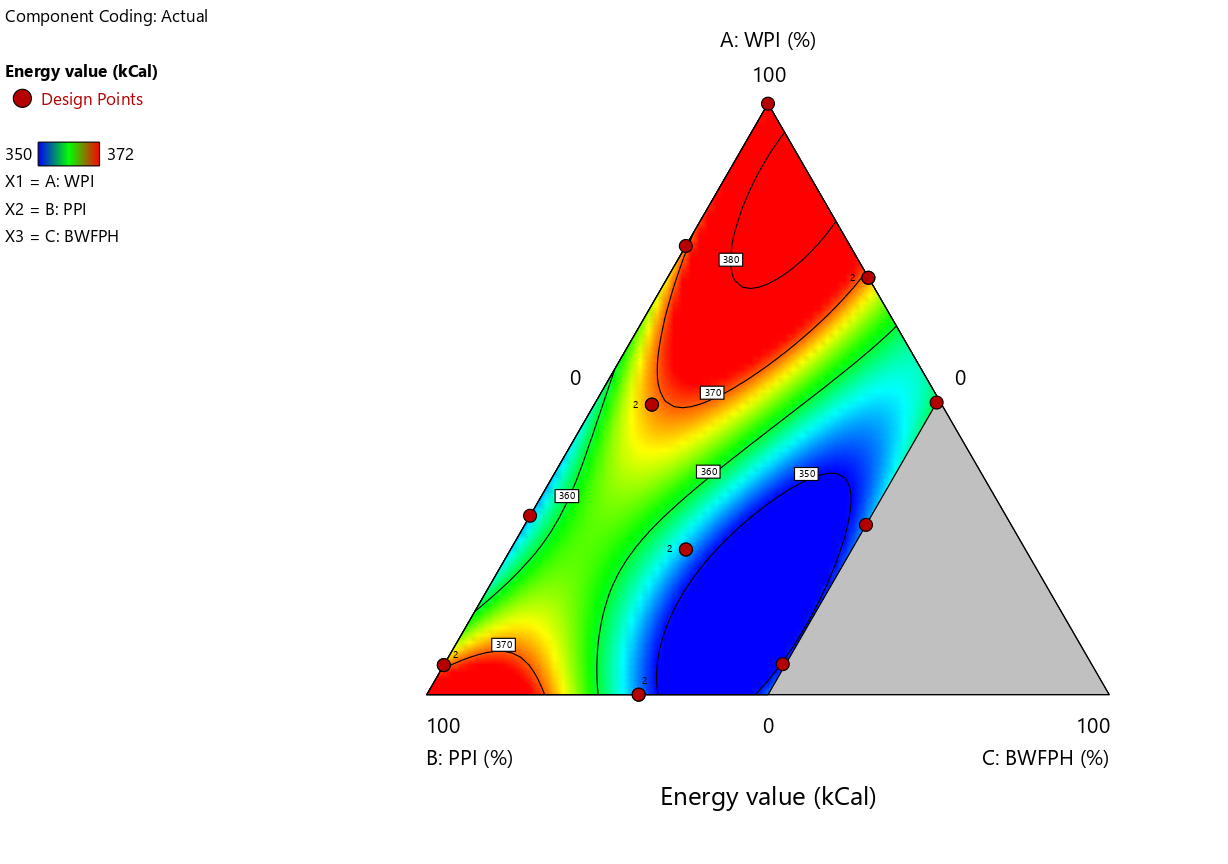  (f) |

**Figure S1. Mixture contour plot of a) Ash content b) Total Carbohydrates c) Fat content d) Moisture content e) Total Protein content f) Energy value of protein bars in relation to different combinations of WPI (A), PPI (B), and BWFPH (C)**

| 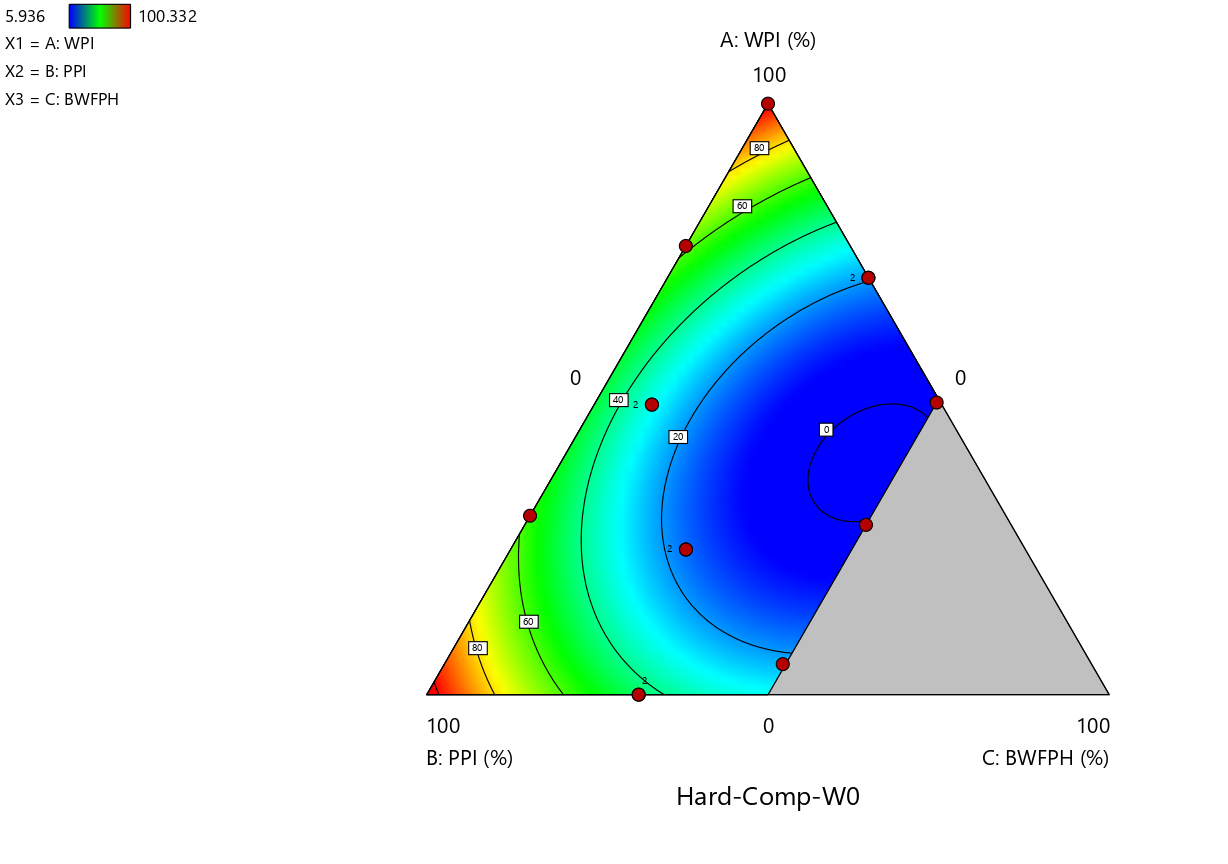  (a) | 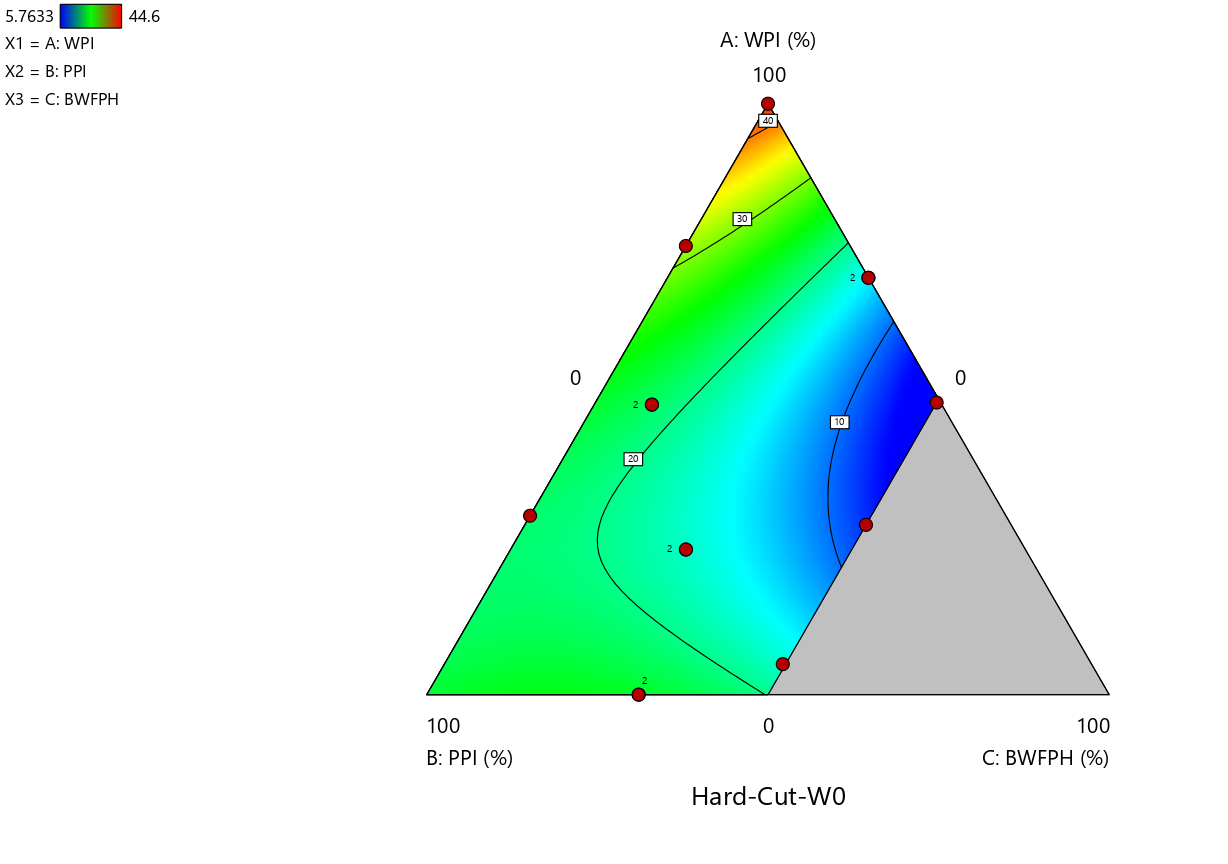  (d) |
| --- | --- |
| 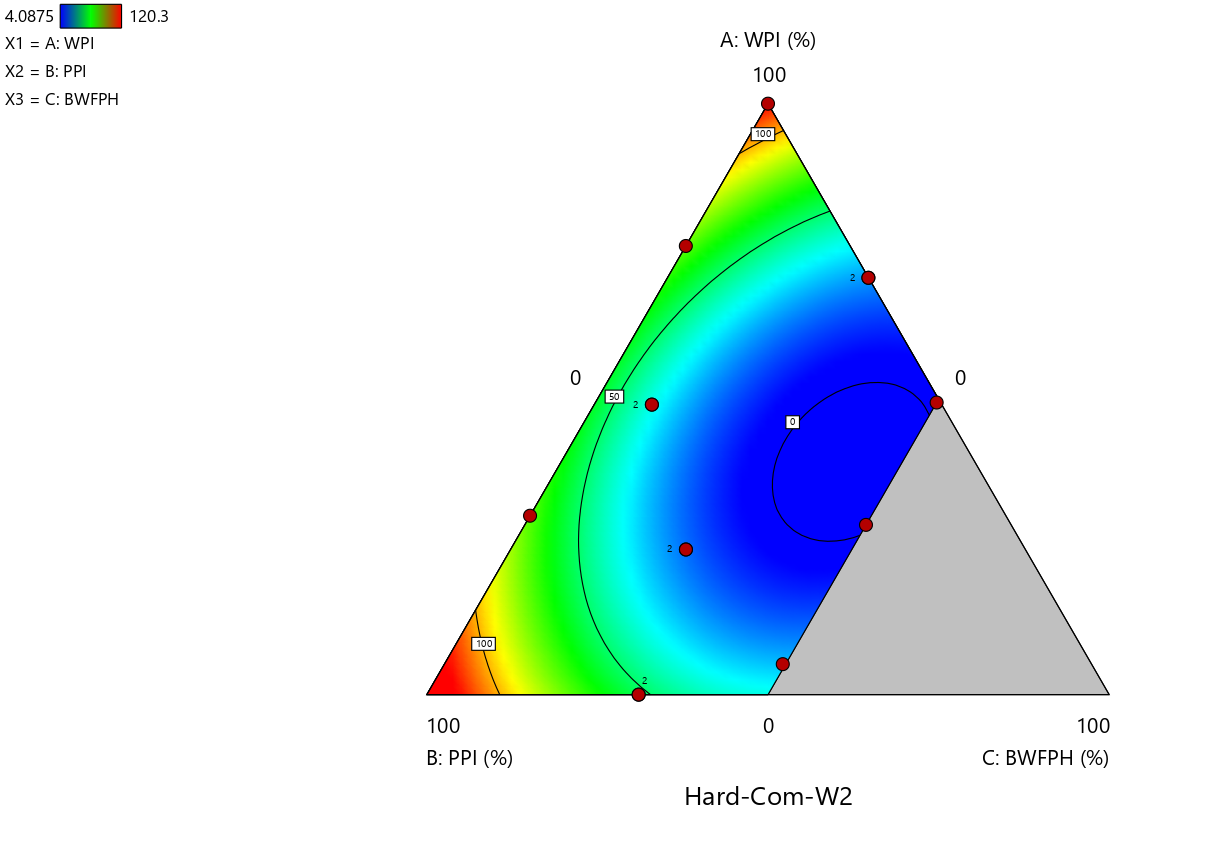  (b) | 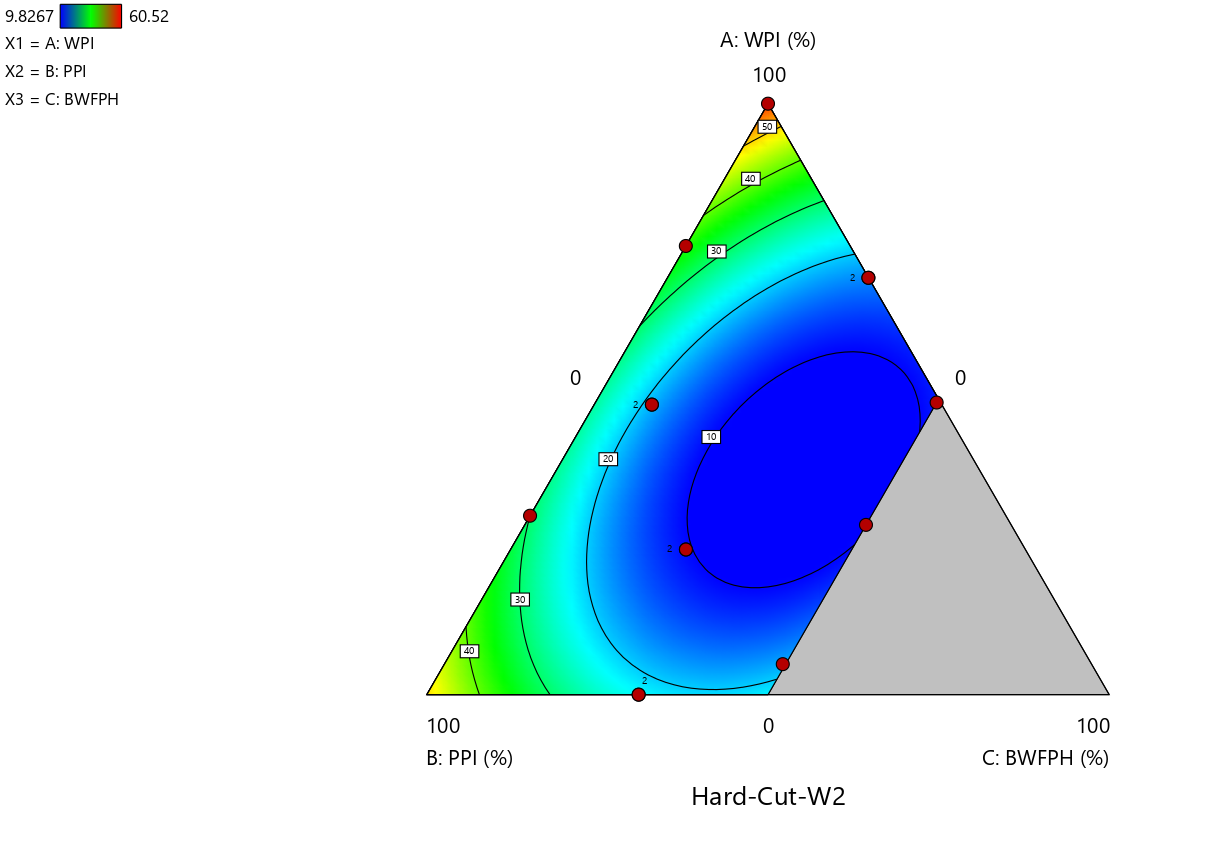  (e) |
| 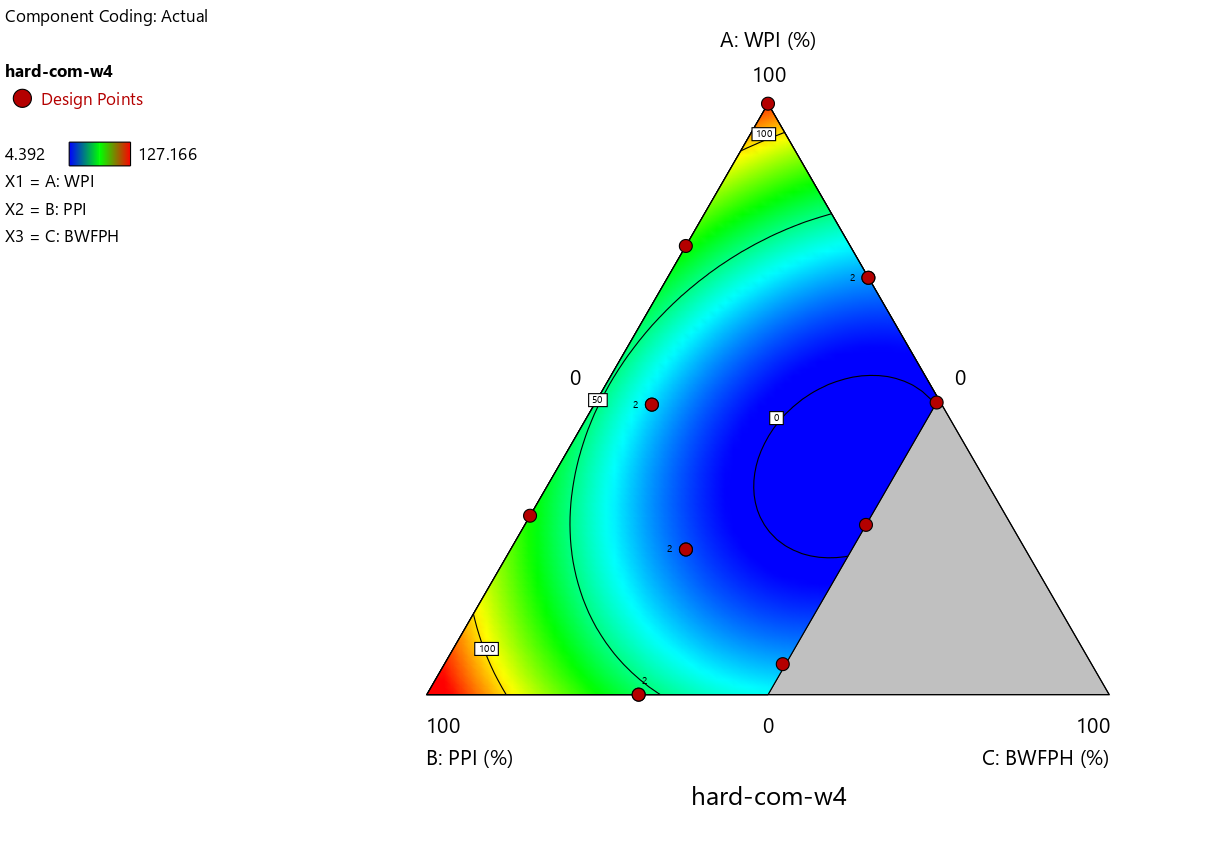  (c) | 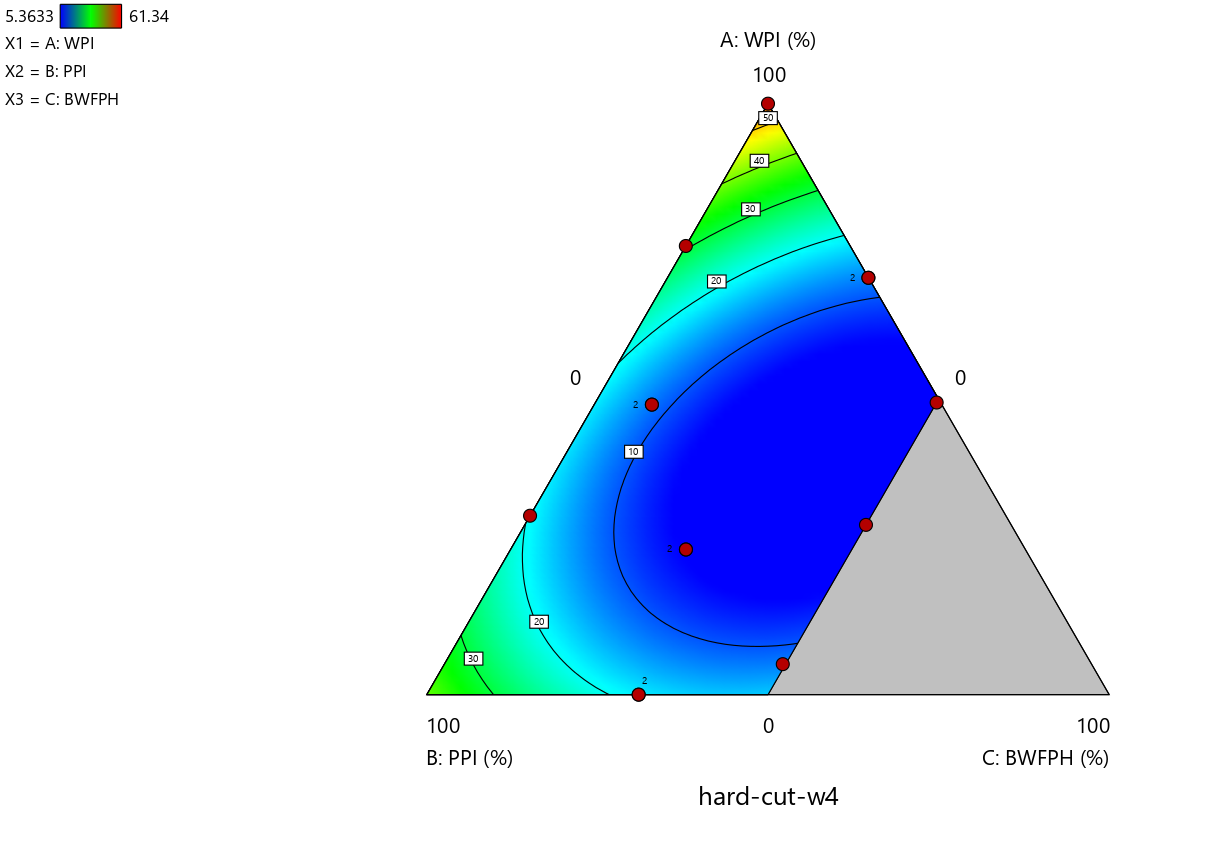  (f) |

**Figure S2. Mixture contour plot of Firmness value (N) at the day 1 (a), day 14 (b), day 28 (c) of storage and Cutting force value (N) in protein bars at the day 1 (d), day 14 (e), day 28 (f) of storage in relation to different combination of WPI (A), PPI (B), and BWFPH (C)**

| 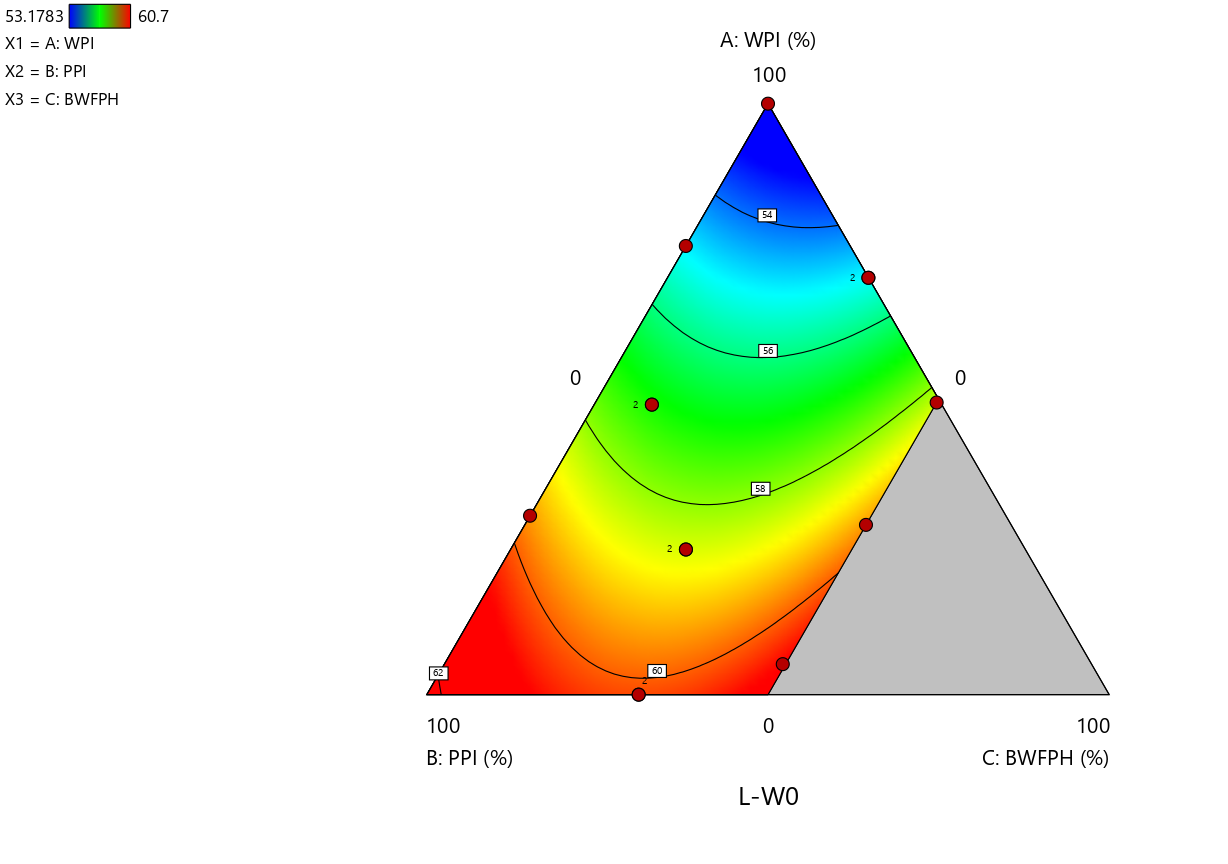  (a) | 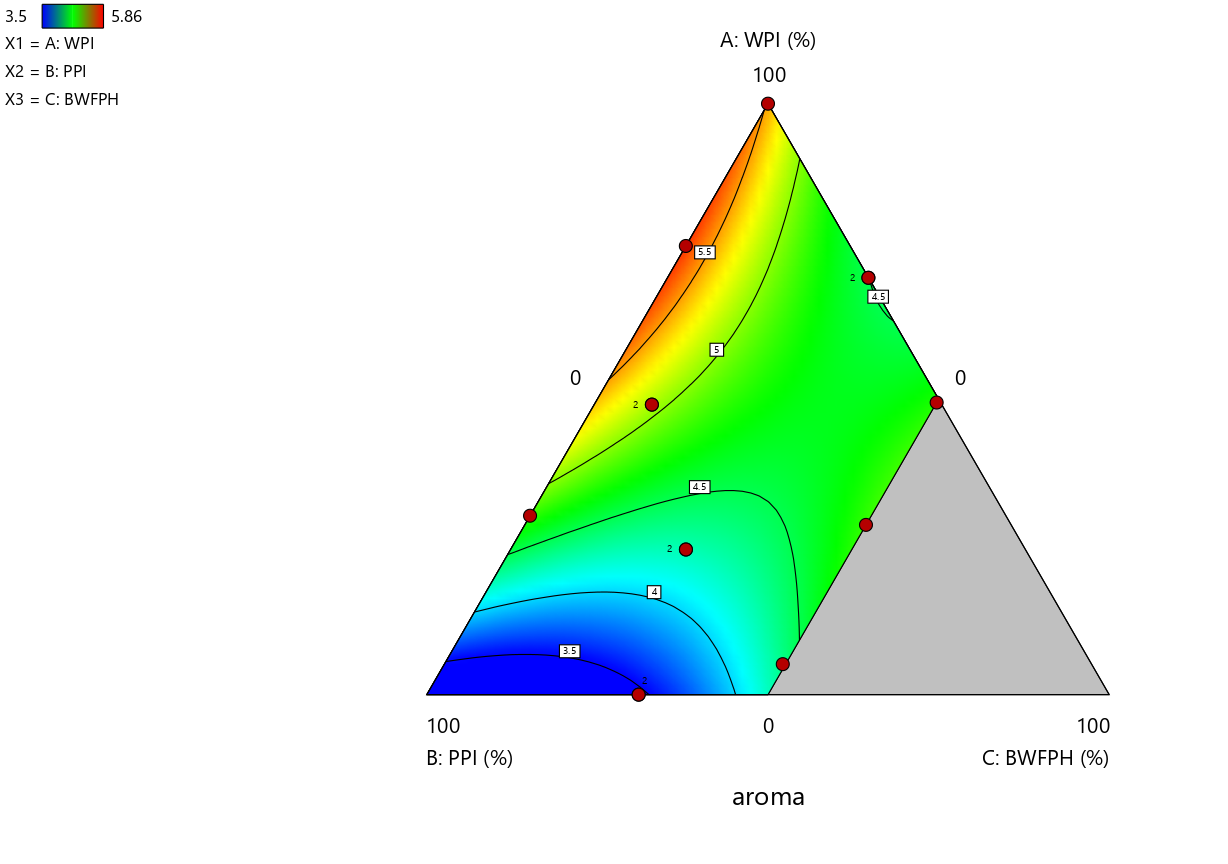  (d) |
| --- | --- |
| 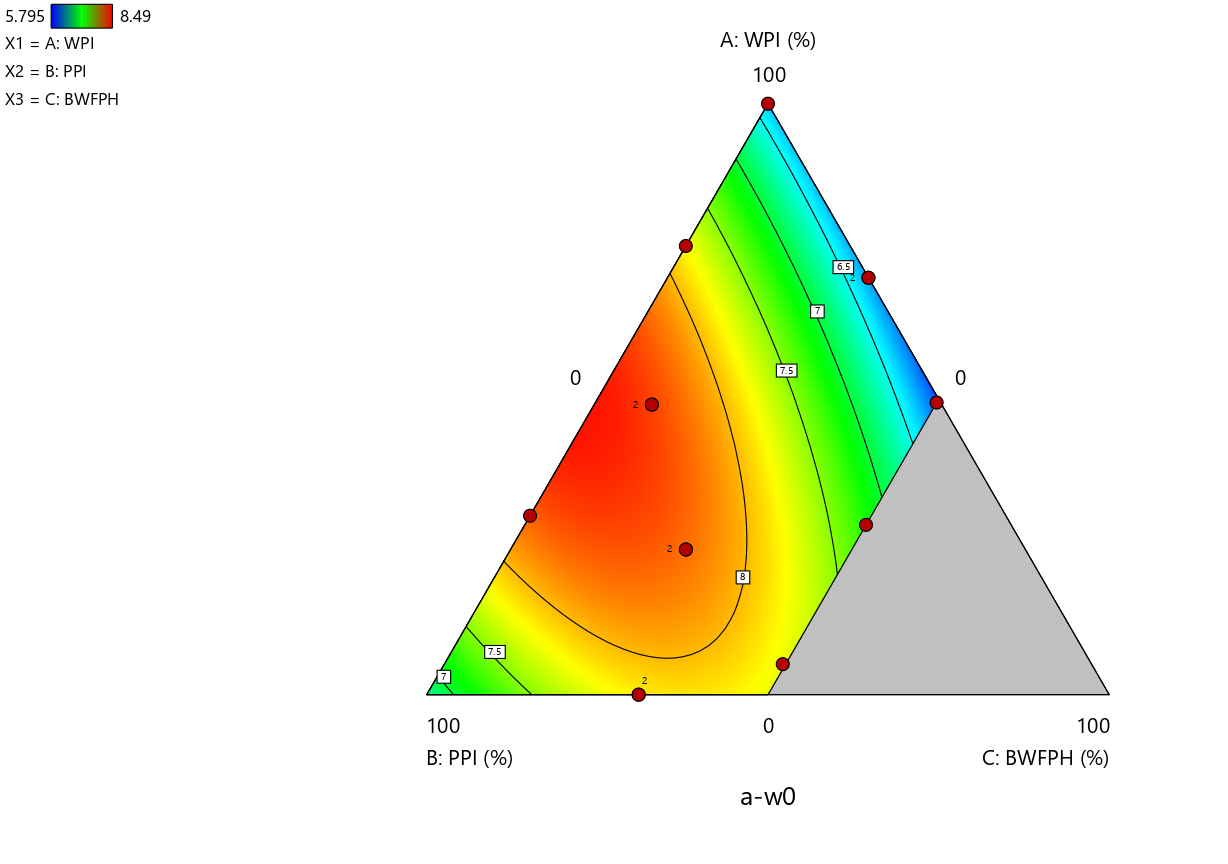  (b) | 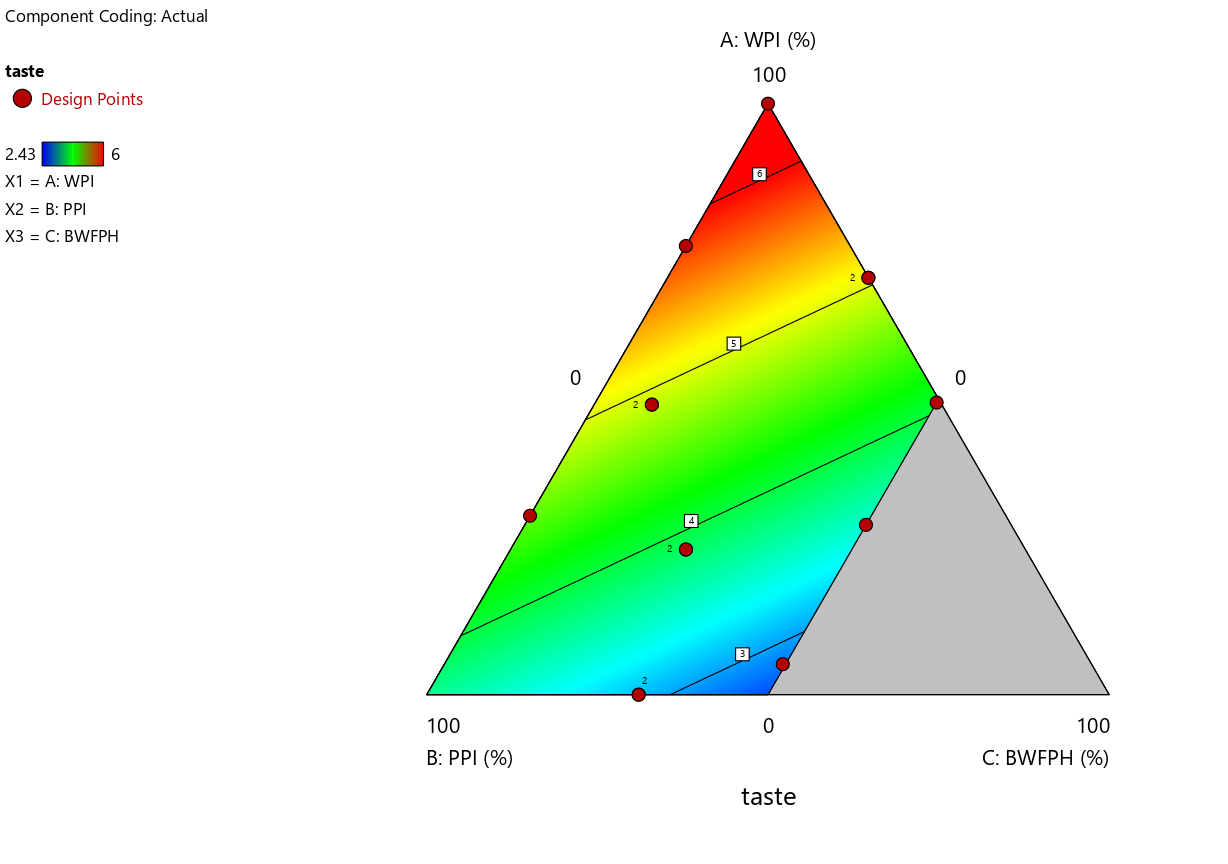  (e) |
| 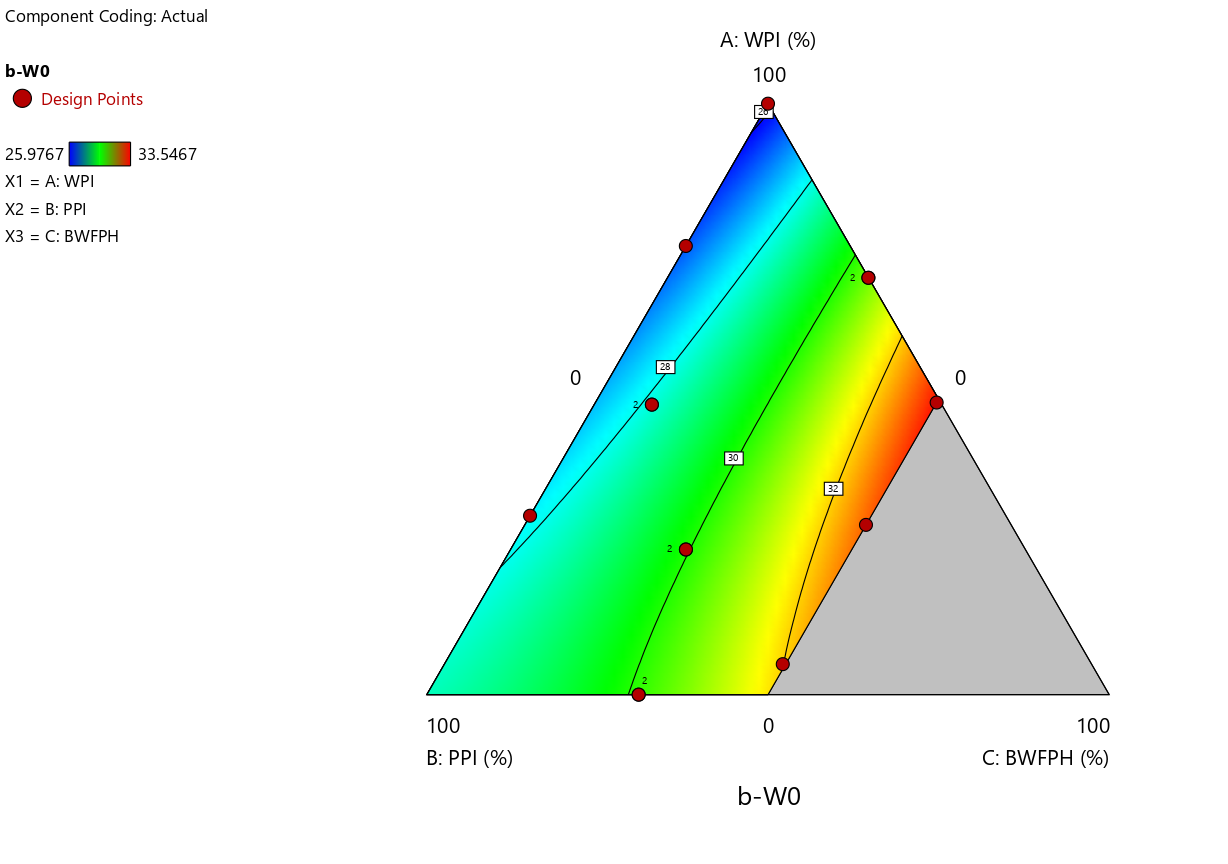  (c) | 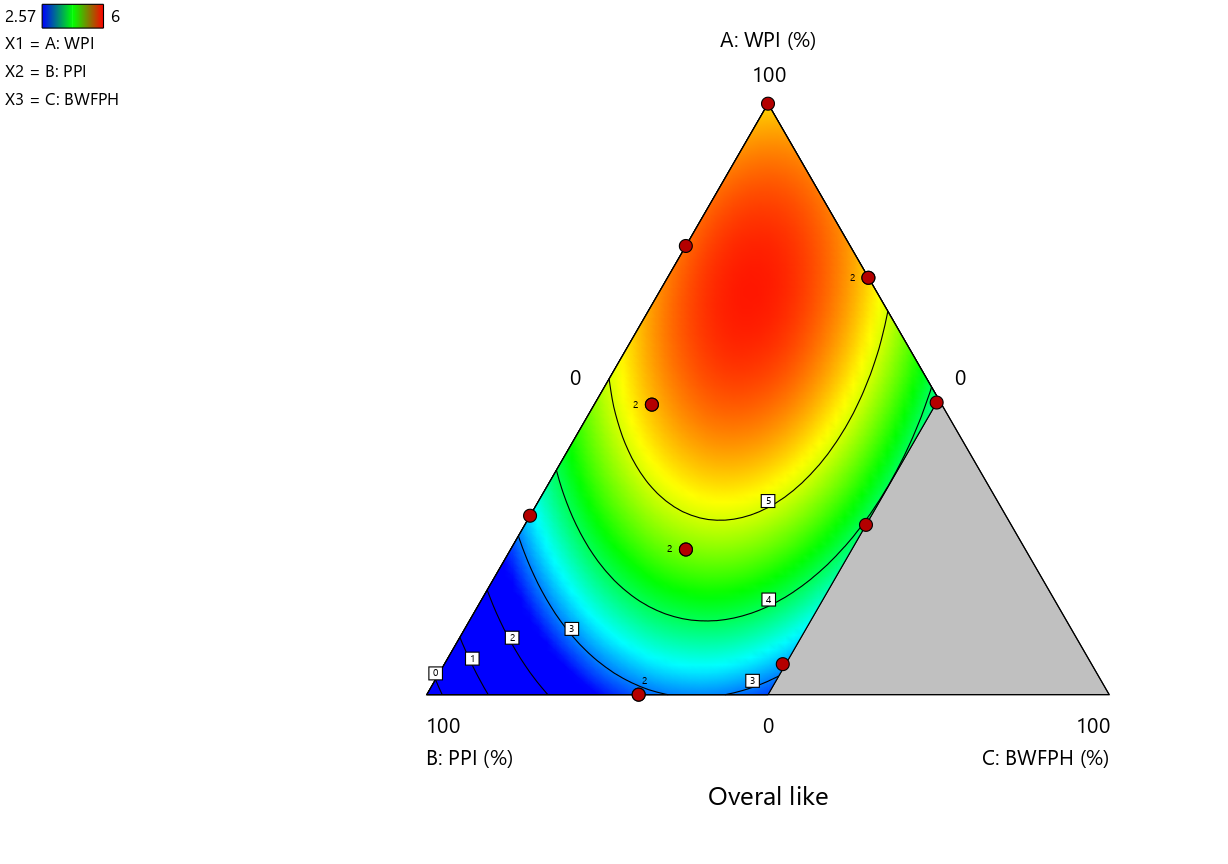  (f) |

**Figure S3. Mixture contour plot of colour parameters: *L** (a), *a**(b), *b** (c) and the sensorial parameters: Aroma (d), taste (e), and overall liking (f) in protein bars in relation to different combination of WPI (A), PPI (B), and BWFPH (C)**


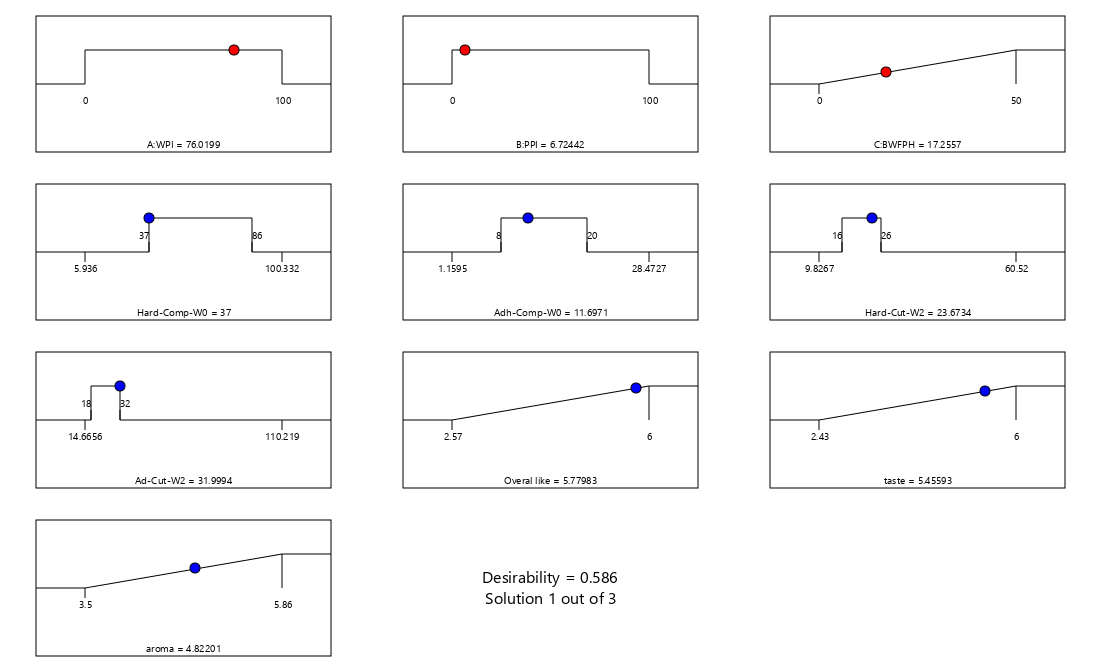


**Figure S4. Response optimization plot for high protein bars prepared from Whey protein isolate (WPI), Pea protein isolate (PPI), and Blue whiting fish protein hydrolysate (BWFPH)**
